# Evaluations on supervised learning methods in the calibration of seven-hole pressure probes

**DOI:** 10.1371/journal.pone.0277672

**Published:** 2023-01-23

**Authors:** Shuni Zhou, Guangxing Wu, Yehong Dong, Yuanxiang Ni, Yuheng Hao, Yunhe Jiang, Chuang Zhou, Zhiyu Tao

**Affiliations:** 1 Southern Marine Science and Engineering Guangdong Laboratory (Zhanjiang), Zhanjiang, China; 2 Guangdong Haizhuang Offshore Windpower Research Center Company Limited, Zhanjiang, China; 3 CSSC Haizhuang Windpower Company Limited, Chongqing, China; 4 School of Renewable Energy, North China Electric Power University, Beijing, China; 5 Institute of Engineering Thermophysics, Chinese Academy of Sciences, Beijing, China; PLOS ONE, UNITED KINGDOM

## Abstract

Machine learning method has become a popular, convenient and efficient computing tool applied to many industries at present. Multi-hole pressure probe is an important technique widely used in flow vector measurement. It is a new attempt to integrate machine learning method into multi-hole probe measurement. In this work, six typical supervised learning methods in scikit-learn library are selected for parameter adjustment at first. Based on the optimal parameters, a comprehensive evaluation is conducted from four aspects: prediction accuracy, prediction efficiency, feature sensitivity and robustness on the failure of some hole port. As results, random forests and K-nearest neighbors’ algorithms have the better comprehensive prediction performance. Compared with the in-house traditional algorithm, the machine learning algorithms have the great advantages in the computational efficiency and the convenience of writing code. Multi-layer perceptron and support vector machines are the most time-consuming algorithms among the six algorithms. The prediction accuracy of all the algorithms is very sensitive to the features. Using the features based on the physical knowledge can obtain a high accuracy predicted results. Finally, KNN algorithm is successfully applied to field measurements on the angle of attack of a wind turbine blades. These findings provided a new reference for the application of machine learning method in multi-hole probe calibration and measurement.

## 1. Introduction

Multi-hole probes (MHPs) which can measure the flow angle and speed of airstreams, are widely used in many fields, such as wind-tunnel and field tests for the aviation [1], automobiles [2], compressors [3] and wind turbines [4–7]. Optical techniques of measuring flow velocity, which are non-contact with the measured fluids, have advantages in obtaining the undisturbed flow. However, they have some limitations in the test conditions and cost considerations. For instance, particle image velocimetry (PIV) is difficult to apply to a larger field of view test, such as field test of large-scale wind turbine. And complex optical adjustments are necessary before achieving accurate flow measurement [8–11]. Doppler LiDAR usually has lower spatial resolution for flow structure measurement [12, 13]. In contrast, multi-hole probes in many cases can provide the easiest-to-use and most cost-effective method for three-velocity-component and pressure measurements in laboratory and industrial environments [14–16]. Moreover, compared to optical techniques, MHPs are more robust and reliable options for industrial applications in harsh working conditions, such as airspeed tubes on the airplane, atmospheric measurements with remotely piloted aircraft [17], and inflow measurements on rotational wind turbine blades [5, 7].

The minimum number of holes that are used for three-dimensional flow measurement is four, while probes with five or seven holes are common and probes with more than ten holes can also be found [18]. With increasing number of holes, the range of the angle of attack that can still be measured increases. There are also probes with only one pressure port, which is constantly turned inside the probe [19]. The shape of the probe (conical, hemispherical or faceted) has an effect on the maximum angle of attack as well as on the sensitivity regarding Reynolds number changes [20], due to the different points of flow separation.

Measurement principle of multi-hole pressure probes is based on the correlation between pressure distributions over a solid surface immersed in the flow and flow angles [21–23]. After measuring the pressure distributions on the surface of the probe head, the flow velocity and angle can be calculated by using the correlation matrix. Therefore, the accuracy of probe measurement is dominated by the correlation matrix calculation. Due to manufacturing errors, it is necessary to calibrate each probe, that is, to obtain the correlation matrix of each probe. It is generally obtained through the following steps [24, 25]. Firstly, port pressures, static pressure and total pressure are collected at the flow conditions whose velocity magnitude and directions are known. The calibration data set is generated. Usually, this procedure is conducted in wind tunnel or free jet nozzle with actuator device adjusting probe attitude angles. Secondly, the pressure data are transformed into appropriate non-dimensional directional coefficients, which are sensitive to the flow variables. The definition of directional coefficients from Krause [26] and Gallington [21] has been widely used in five-hole probe and seven-hole probe calibration, respectively. Pisasale and Ahmed [27] proposed a new normalization technique to extend the calibration range of five-hole probe. Paul [28] defined the different non-dimensional pressure coefficients to obtain less computational errors. Finally, the velocity magnitude and direction of an unknown flow field will be calculated from comparisons between the measured port pressures and the calibration data set. The calculation methods include direct-interpolation developed by Treaster and Yocum [29] and Zilliac [22, 23], least squares polynomial curve-fit developed by Gallington [21, 30], look-up error tables developed by Wenger [31], and neural networks developed by Rediniotis [32–35].

Machine learning method, as an independent research topic, has been developed since the 1940s. The most representative work is the M-P neuron model proposed McCulloch and Pitts in 1943 [36]. After that, more and more machine learning models are developed to help solve the problems at data analysis and decision making [37–40]. They are widely used in a growing number of fields today including computer vision, natural language processing, and medical diagnosis [41]. Machine learning algorithms can be divided into supervised learning, unsupervised learning and reinforcement learning. Actually, calibration of seven-hole pressure probes is a classic supervised learning process. The recorded port pressures in known flow velocity magnitude and directions can be split in to the training dataset and test dataset. However, only few literatures [32–35] conducted the probe calibrations with machine learning method, and only artificial neural networks were focused.

In 2012, a Python module, *Scikit-learn*, integrating a wide range of state-of-the-art machine learning algorithms for medium-scale supervised and unsupervised problems was proposed. This package focuses on bringing machine learning to non-specialists using a general-purpose high-level language [42]. Based on *Scikit-learn*, the machine learning algorithms become a user-friendly toolkit, especially for parametric study. Supervised problems include regression and classification problems [38]. The calibration of seven-hole pressure probes belongs to regression problem. There are lots of regression algorithms in *Scikit-learn*, such as Nearest Neighbors, Polynomial regression, Neural network, Support Vector Machines, Decision Trees and Random Forests.

Are the machine learning methods suitable for seven-hole probe calibration? What are the advantages and disadvantages of the above algorithms in the seven-hole pressure probe calibrations? Which algorithm is the most suitable in comparison?

To answer these questions, six typical supervised learning methods were chosen for the seven-hole probe calibration. The contributions of this work mainly lie in two aspects. First, the performance of the supervised learning methods in the probe calibration is systematically evaluated from prediction accuracy, computational time, feature sensitivity, and robustness on the failure of some hole port. Second, compared with the in-house traditional algorithm, Random forests and K-nearest neighbors algorithms have the great advantages in the computational efficiency and the convenience of writing code with the same prediction accuracy. This shows that the supervised learning methods has great potential in real-time online calibration of multi-hole probes.

## 2 Calibration data set from wind tunnel measurement

**Fig 1:**
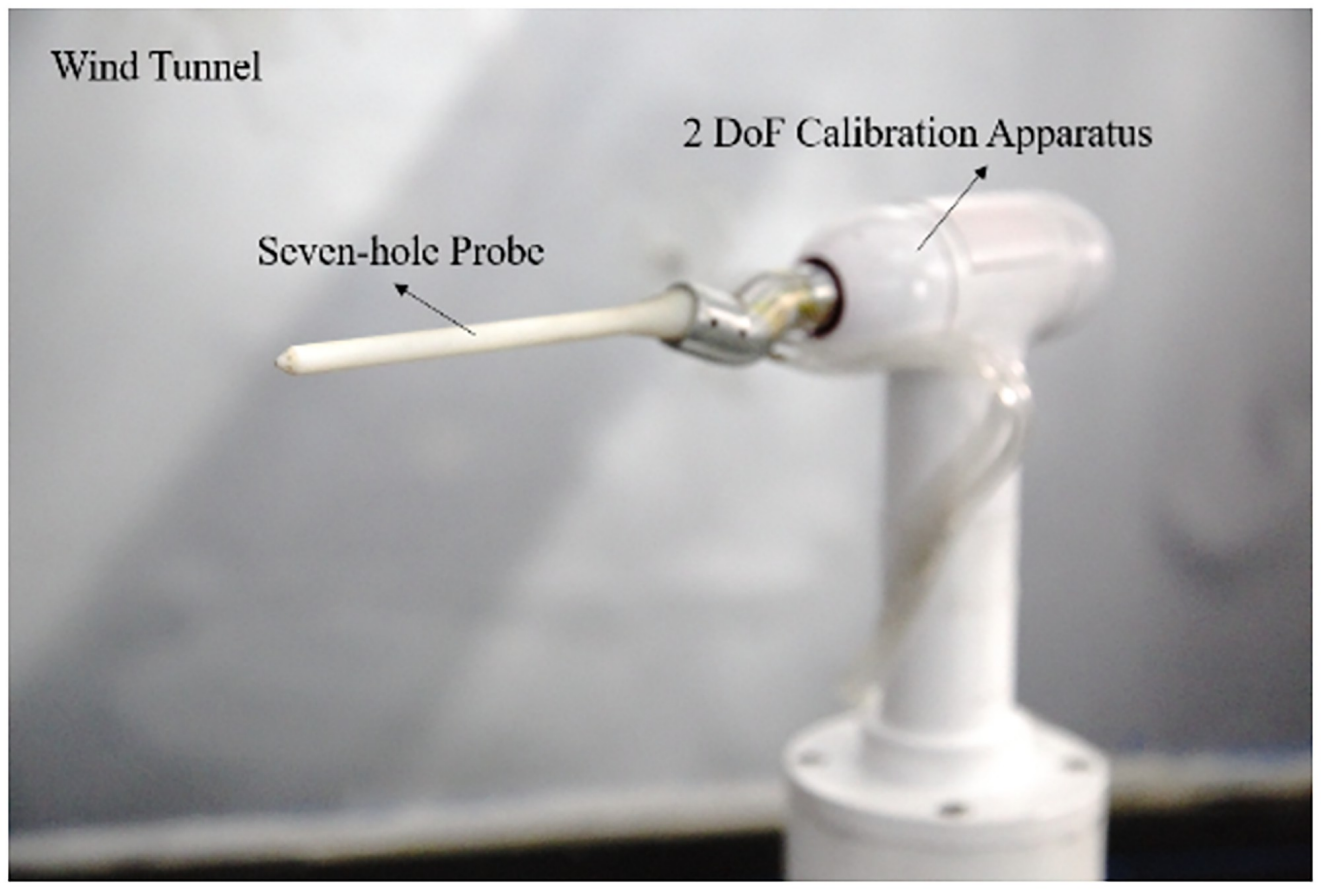
The experimental setup on the seven-hole probe calibration.

The seven-hole probe tested in this work was manufactured by 3-D printing techniques with manufacturing accuracy of 0.01mm. The calibration measurements were carried out in a closed test section of a wind tunnel with an outlet size of 0.5 m by 0.5 m. The turbulence intensity is less than 0.2% when the free stream wind speed is 20 m/s. The probe was installed on a two degrees of freedom calibration apparatus, which includes an electric rotator, a lifting platform and a customized rolling device. The experimental setup on the seven-hole probe calibration is shown in Fig 1. The detailed parameters of the experimental setup can be found in the authors’ publication [25].

**Fig 2:**
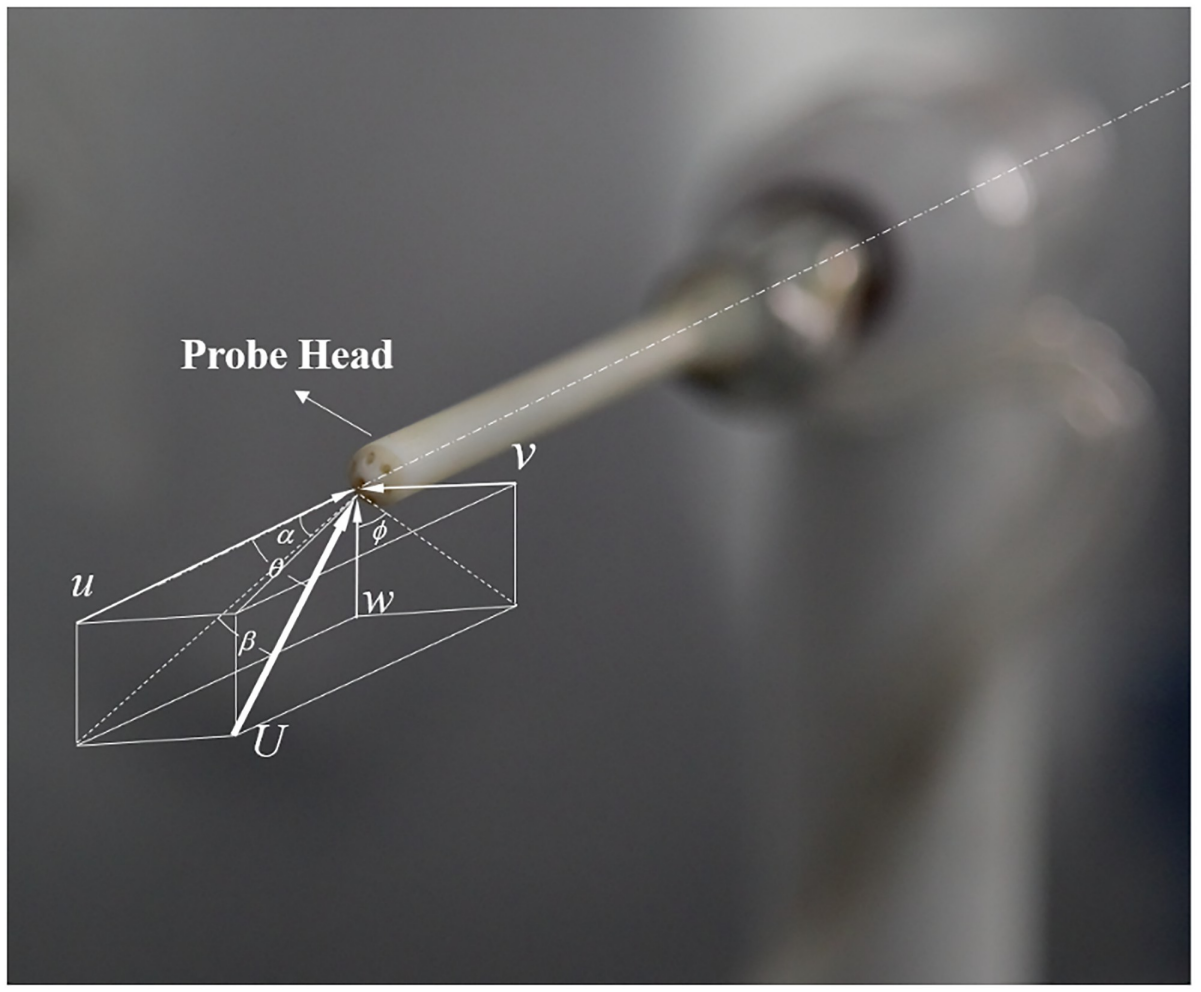
The body-coordinate system of probe and flow angles definitions.

The body-coordinate system is defined as Fig 2, in which *x*-axis is parallel to the axis of probe tip and points to probe tail, *z*-axis points from Port 7 to Port 1, and *y*-axis meets the right-hand coordinate system. Two flow angle systems are discussed. One is *α*-*β* system, where angle of attack *α* is the angle between the probe’s *x*-axis and the projection of the velocity vector in the vertical plane, and sideslip angle *β* is defined as the angle between the velocity vector and the vertical plane. The other is *θ*-*ϕ* system, where pitch angle *θ* is the absolute angle between the velocity vector and the probe’s *x*–axis, and roll angle *ϕ* describes the azimuthal orientation of the velocity vector in the *y*-*z* plane, measured positive counterclockwise from the negative *z*-axis as viewed from the front. The relationships between the two angle system definitions are as Eq (1).


$$\left\{\begin{array}{l} \alpha =\text{arctan}\frac{w}{u}=\text{arctan}\frac{U\;\text{sin}\;\theta \;\text{cos}\;\phi }{U\;\text{cos}\;\theta }=\text{arctan}(\text{tan}\;\theta \;\text{cos}\;\phi )\\ \beta =\text{arcsin}\frac{v}{U}=\text{arcsin}\frac{U\;\text{sin}\;\theta \;\text{sin}\;\phi }{U}=\text{arcsin}(\text{sin}\;\theta \;\text{sin}\;\phi ) \end{array}\right.$$ (1)

**Fig 3:**
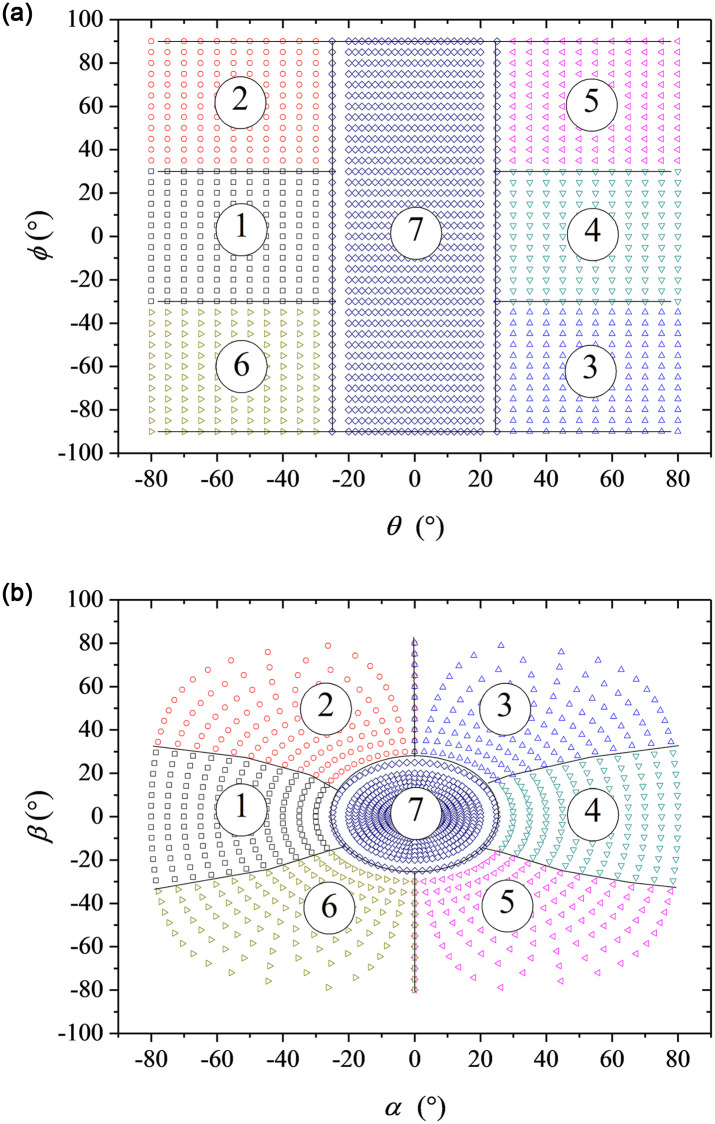
Angular range of calibration database, θ~ϕ coordinates (a), α~β coordinates (b).

Angular range of calibration data set shown in Fig 3 is from -80° to 80° for pitching angle and from -90° to 90° for rolling angle. The interval of pitching angle is 2° from -20° to 20°, and 5° from ±20° to ±80°. The interval of rolling angle is 5°. Wind speeds of freestream in calibration dataset are 10 m/s, 20m/s and 30 m/s. Therefore, the data set is a 4995 ×10 matrix. The 4995 rows of data include the combination parameter of 3 wind speeds, 45 pitch angles, and 37 roll angles. The 10 columns of data represent wind speed, pitch angle, roll angle, and 7 port pressures, respectively. The table header in the attached data file shows the variables and units corresponding to each column of data. In the machine learning algorithms, the data at 10 m/s and 30m/s are as training set, and the data at 20 m/s and pitching angle from -50° to 50° are as test set.

Port number with highest pressure was recorded as dominant partition at each flow angle. Based on this definition, the dominant partition distribution of the database is obtained and indicated in *θ*-*ϕ* coordinate and *α*-*β* coordinate in Fig 3, where the black line is theoretical boundary to divide the seven partitions. The partitions are conducive to understanding the distribution of prediction errors.

## 3 Calibration process

**Fig 4:**
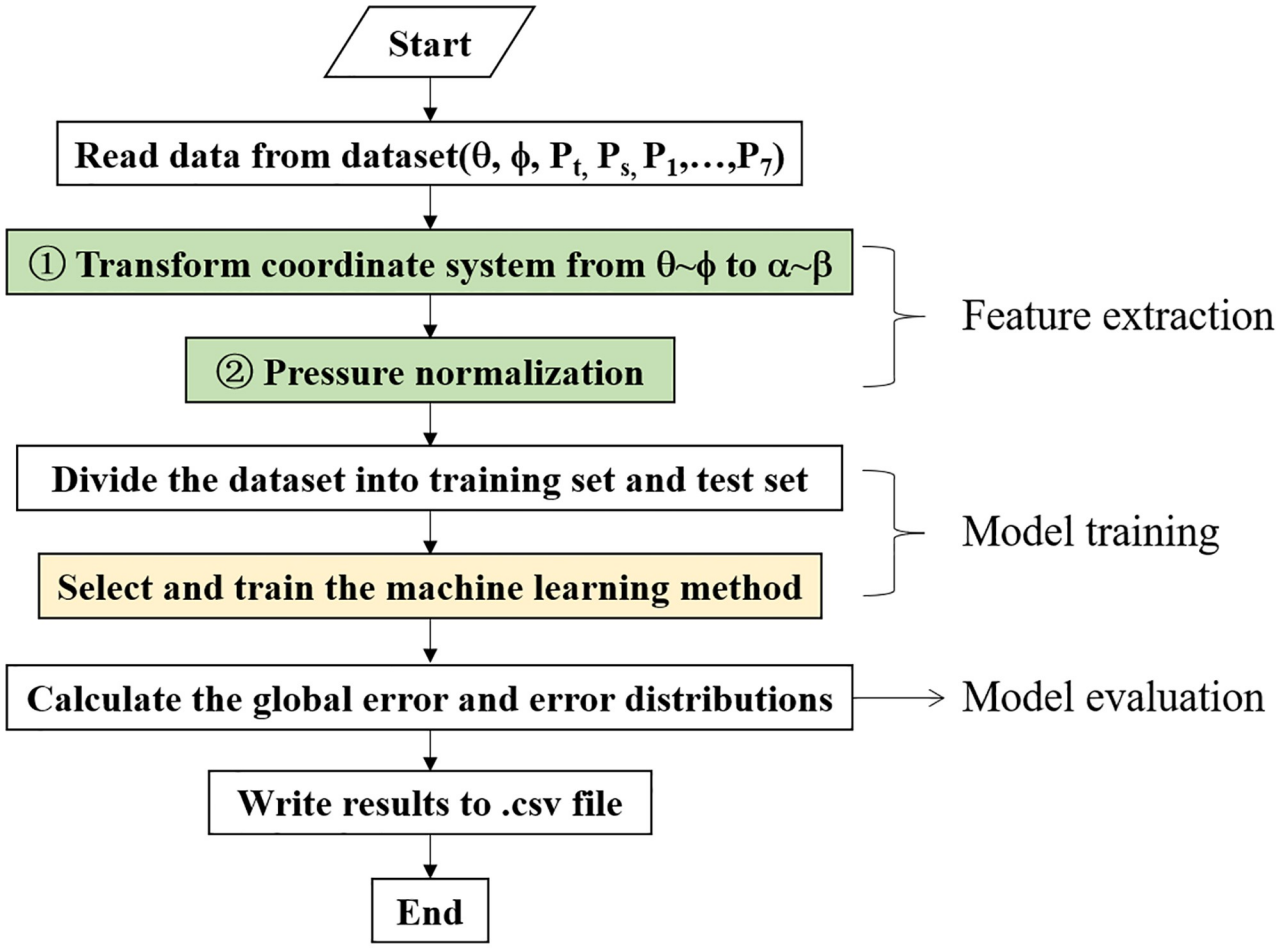
Basic flow chart of the seven-hole probe calibration with machine learning methods.

The probe calibration is the process finding out the correlation between port pressure distributions and flow parameters including flow angles and velocity. The flow chart of the seven-hole probe calibration with machine learning methods is shown in Fig 4. Basically, the process includes three parts: feature extraction, model training, and model evaluation. The purpose of feature extraction is to find out the most suitable features for the machine learning model. In the Python module, *Scikit-learn*, the machine learning model can be called directly, which provides the convenience for the non-specialists, and the calibration codes are simple and compact.

**Fig 5:**
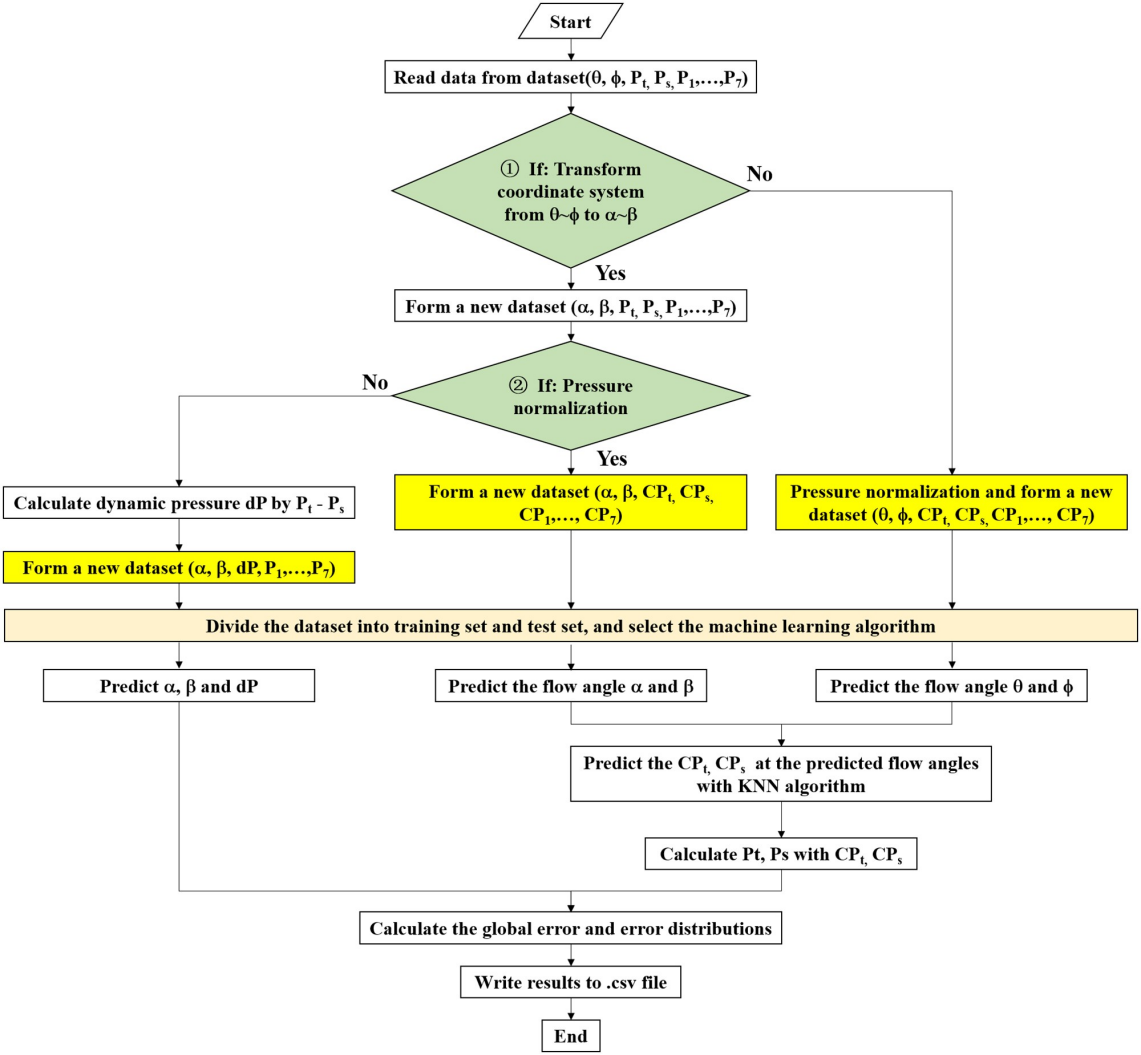
Flow chart of probe calibration with three different feature extraction methods.

Usually, feature extraction is time-consuming and plays an important role in the prediction accuracy of the machine learning models. With regard to seven-hole probe calibration, machine learning methods are used to find out the correlation between port pressure distributions and flow parameters. In the dataset of probe calibration, the features include *θ*, *ϕ*, *p*_*t*_, *p*_*s*_, *p*_1_,…,*p*_7_, where *p*_*t*_ and *p*_*s*_ are the total and static pressure representing flow velocity, *p*_1_,…,*p*_7_ are the pressure of seven ports, respectively. In most of the traditional calibration methods, the data fitting algorithms are developed to find out the correlation between flow angles *α*, *β* and normalized pressure values. This is due to normalized pressure values or pressure coefficients have the direct relationship with flow angle *α*, *β* based on the aerodynamic theory [25]. However, as a mathematical technology, machine learning can still work without the physical knowledge. Therefore, the effects of feature extraction on the prediction accuracy are focused on in this work to investigate the feature sensitivity of machine learning methods. Based on the coordinate system of flow angles and normalization of pressure values, the relationship of features can be expressed in three types, as shown in Eqs (2–4).

$$\left(\alpha ,\beta \right)=f\left(Cp_{1},\ldots ,Cp_{7}\right)$$ (2)

$$\left(\theta ,\phi \right)=f\left(Cp_{1},\ldots ,Cp_{7}\right)$$ (3)

$$\left(\alpha ,\beta ,dp\right)=f\left(p_{1},\ldots ,p_{7}\right)$$ (4)

where, $Cp_{i}=\frac{P_{i}-\left(\sum \left(P_{1},P_{2},\ldots ,P_{7}\right)-\text{max}\left(P_{1},P_{2},\ldots ,P_{7}\right)\right)/6}{\text{max}\left(P_{1},P_{2},\ldots ,P_{7}\right)-\left(\sum \left(P_{1},P_{2},\ldots ,P_{7}\right)-\text{max}\left(P_{1},P_{2},\ldots ,P_{7}\right)\right)/6}$, which is a pressure normalization method proposed in the authors’ previous work [25], and *d*_*p*_ = *p*_*t*_ − *p*_*s*_. In Eq (2), the features are flow angles *α*, *β* and normalized pressure values. In Eq (3), the features are flow angles *θ*, *ϕ* and normalized pressure values. In Eq (4), the features are flow angles *α*, *β* dynamic pressure and dimensional pressure values. The flow chart of calibration process with the three feature extraction methods, which are highlighted in yellow background, is shown in Fig 5. For feature extraction methods in Eqs (2) and (3), normalized pressure values are irrelevant to the flow velocity or dynamic pressure, so only the flow angles were predicted firstly. Then a secondary prediction on the total and static pressures was conducted with K- nearest neighbors.

Based on the above calibration process, parameter of each machine learning algorithm was adjusted and optimized firstly based on the global errors. The global error is computed by root mean square error (RMSE), which is defined as Eq (5). where m is the number of test set, *h*(*x*_*i*_) is the predicted result at the input point of *x*_*i*_, and *y*_*i*_ is the output truth value.


$$\text{RMSE}=\sqrt{\frac{1}{m}\sum_{i=1}^{m}{\left(h\left(x_{i}\right)-y_{i}\right)}^{2}}$$ (5)

Then all the machine learning algorithms with optimal parameters were evaluated in comparison from prediction accuracy, computational time, feature sensitivity and robustness on the failure of some hole port.

## 4 Parameter adjustment based on the RMSEs

The regression algorithms this work focused on include K-nearest neighbors, polynomial regression, multi-layer perceptron, support vector machines, decision trees and random forests. The detailed illustrations on these algorithms can be found in the user guide of *Scikit-learn*, which will not be repeated in this work [42]. The key parameters of each algorithms were tested firstly. In this section, only features in Eq (2) were selected. The RMSEs are calculated from all the error data in the test set.

### 4.1 K-nearest neighbors

K-nearest neighbor algorithm is to find a predefined number of training samples closest in distance to the new point, and predict the label from these training samples [43]. The parameters of K-nearest neighbor algorithm in *Scikit-learn* module include number of neighbors, weights, algorithm, power parameter for the Minkowski metric. In this work, the effects of number of neighbors and weights are studied. Power parameter is chosen to be 2 and algorithm is automatically selected from BallTree, KDTree and brute-force search.

**Fig 6:**
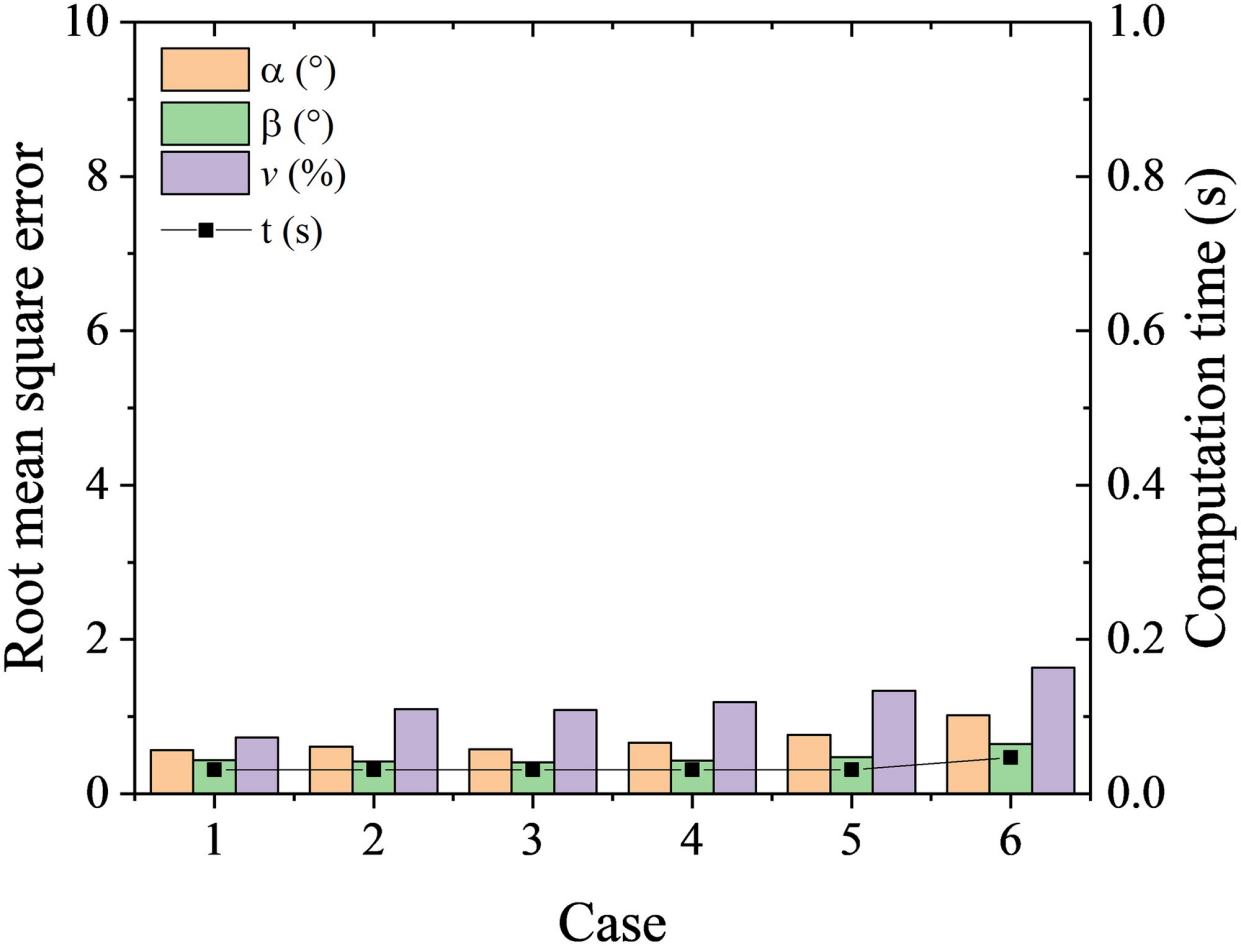
RMSEs and computation time at different cases with K-nearest neighbors.

Six cases with different parameters in Table 1 were tested. It is shown from Fig 6 number of neighbors has slight effects on the RMSEs. Overall, the RMSEs of flow angles *α*, *β* first increase then decrease with the increase of number of neighbors. A better calibration is obtained when using weight of distance by comparison from case 3 and 6. The best calibration results are RMSEs of 0.6° for *α*, 0.4° for *β* and 1% of *v*. The computation time at all the cases is less than 0.1s, which shows the K-nearest neighbor algorithm has very high efficiency.

**Table 1:** Cases at different parameter setups.

Case	1	2	3	4	5	6
**Number of neighbors**	1	3	5	15	25	5
**weights**	distance	distance	distance	distance	distance	uniform

### 4.2 Polynomial regression

**Fig 7:**
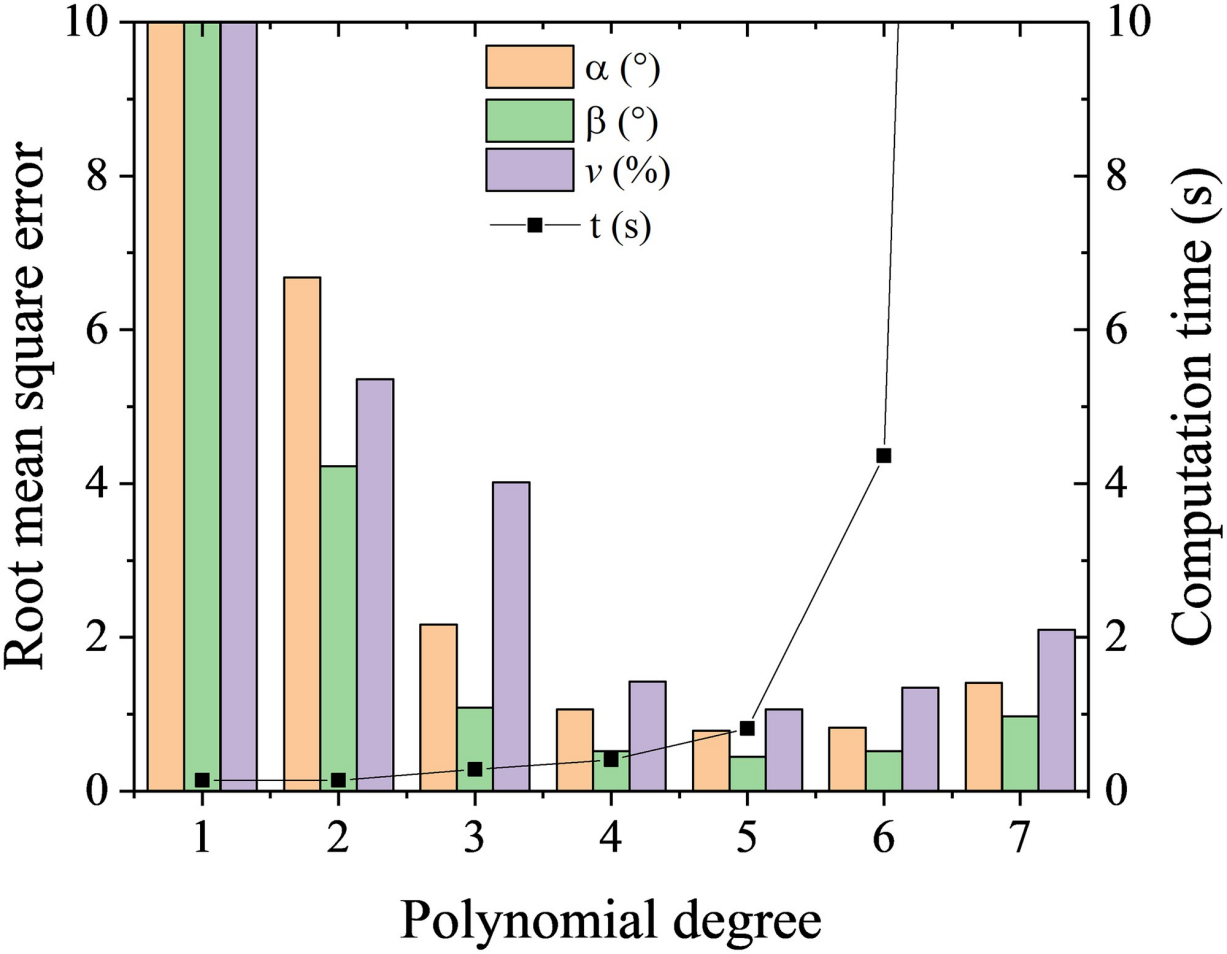
RMSEs and computation time at different cases with polynomial regression.

Polynomial regression fits a polynomial model with coefficients to minimize the residual sum of squares between the observed targets in the dataset based on the least-square method. When the polynomial degree is 1, the polynomial regression degenerate into linear regression. The cases with polynomial degree from 1 to 7 were tested. It is shown from Fig 7 the cases with polynomial degree of 4, 5, and 6 have acceptable predicted accuracy, while the computation time increases exponentially when polynomial degree is larger than 5. Hence polynomial regression with degree of 5 is most suitable for the calibration.

### 4.3 Multi-layer perceptron

Multi-layer perceptron is a supervised learning algorithm that learns a non-linear function approximator for regression. Between the input and the output layer, there can be one or more hidden layers and non-linear activation function [44]. The hidden layer sizes and activation are the most important parameters to determine the performance of multi-layer perceptron. The solver is ‘adam’. L2 penalty (regularization term) parameter is 0.0001. The learning rate is constant and the initial learning rate is 0.001.

**Fig 8:**
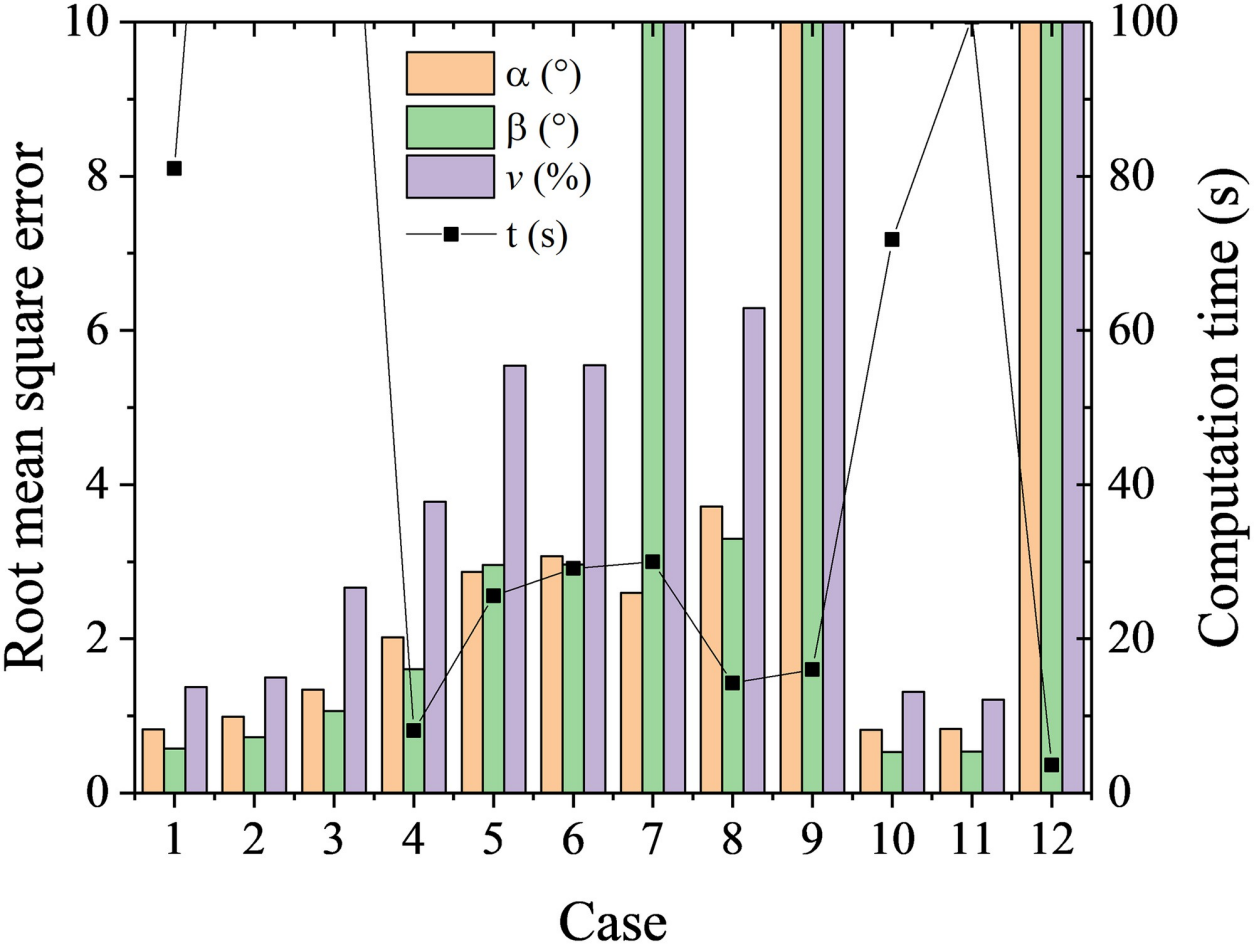
RMSEs and computation time at different cases with multi-layer perceptron.

As shown in Table 2, 12 cases were tested. The results from case 1 to 9 are used to identify the effects of hidden layer sizes, and the results from case 10 to 12 and case 1 are used to identify the effects of activation. It is shown from Fig 8 the first layer size plays a dominant role in the predicted accuracy and computation time. Meanwhile, the predicted accuracy and computation time are not sensitive to the activation functions except for ‘identity’. It is noteworthy that multi-layer perceptron is a time-consuming learning method, which takes time of two orders of magnitude more than K-nearest neighbor algorithm.

**Table 2:** Cases at different parameter setups.

Case	1	2	3	4	5	6	7	8	9	10	11	12
**Hidden layer sizes**	(50,30)	(100)	(50)	(10,6)	(10,6,2)	(10,2)	(6,2)	(6,)	(3,)	(50,30)	(50,30)	(50,30)
**Activation**	relu	relu	relu	relu	relu	relu	relu	relu	relu	tanh	logistic	identity

### 4.4 Support vector machines

Support vector regression is extended from support vector classification. In the support vector regression, the inner product in high dimensional space is skillfully solved by introducing kernel function, and the nonlinear regression problem is well realized [45]. The selection of the kernel function parameter and error penalty factor affected the precision of the support vector machine significantly. Kernel coefficient is default as ‘scale’.

**Fig 9:**
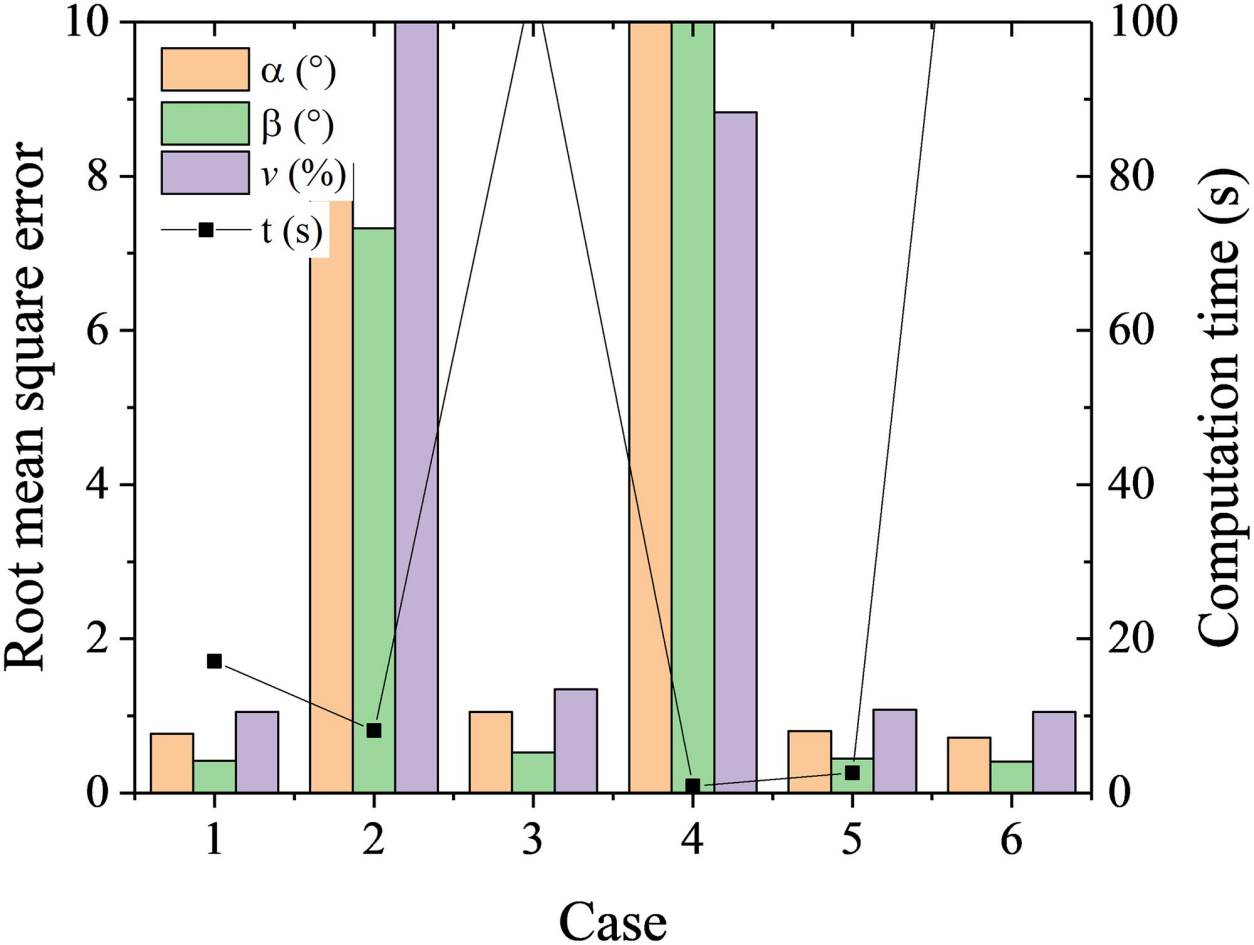
RMSEs and computation time at different cases with support vector machines.

Six cases with different parameters in Table 3 were tested. The results from case 1 to 4 are used to identify the effects of kernel function, and the results from case 5 to 6 and case 1 are used to identify the effects of error penalty factor of C. It is shown from Fig 9 that kernel function has significant influence on the predicted accuracy and computation time. In comparisons among cases from 1 to 4, kernel of ‘rbf’ has best learning performance for seven-hole probe calibration. Error penalty factor of C has little effects on the accuracy but affects the computation time significantly.

**Table 3:** Cases at different parameter setups.

Case	1	2	3	4	5	6
**kernel**	rbf	linear	4^th^ poly	sigmoid	rbf	rbf
**C**	10^3^	10^3^	10^3^	10^3^	10^2^	10^4^

### 4.5 Decision trees and random forests

Decision trees are a kind of tree structure, in which each internal node represents a decision on an attribute, each branch represents the output of a judgment result, and finally each leaf node represents a classification or regression result [46]. Maximum depth is the distance between the node and the root of decision tree. When the depth reaches the specified number, the splitting will be stopped. The criterion is chosen to be ‘squared_error’. The minimum number of samples required to split an internal node is 2. The minimum number of samples required to be at a leaf node is 1.

Random forests can achieve a reduced variance by combining diverse decision trees [47]. The number of estimators represents the number of trees in the forest, which is a main parameter in random forests. The maximum depth of the tree is chosen to be ‘none’, which means nodes are expanded until all leaves are pure or until all leaves contain less than the minimum number of samples required to split an internal node.

**Fig 10:**
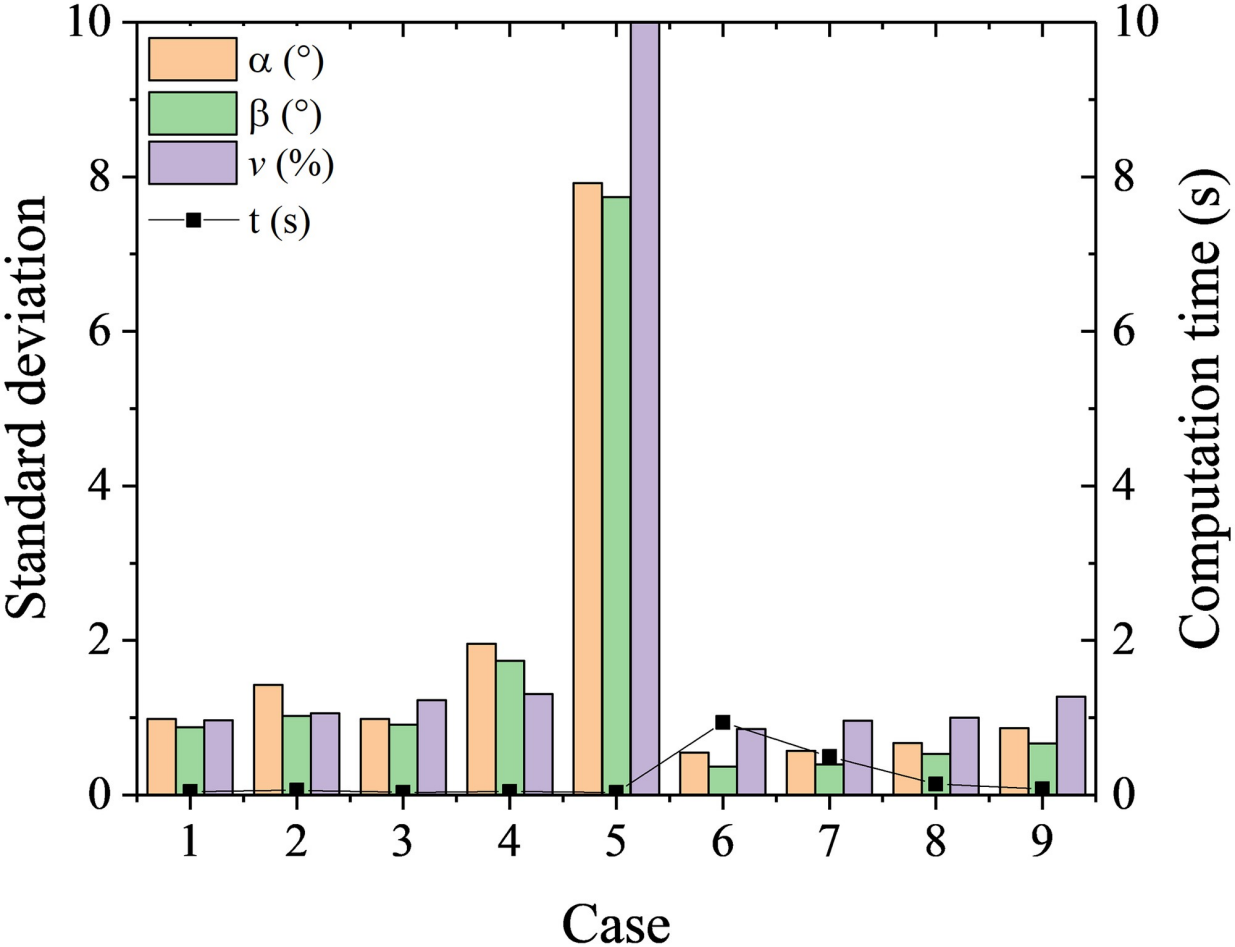
RMSEs and computation time at different cases with decision trees and random forests.

Nine cases with different parameters in Table 4 were tested. It is shown from Fig 10 random forests achieve better calibration accuracy but take slightly more computation time than decision trees overall. In the decision trees, the calibration accuracy stops getting significantly better with max depth of decision trees larger than 20. The computation time is insensitive to the max depth. In the random forests, with the increase of number of estimators, the RMSEs of flow angles decrease, and the computation time increases in second-level.

**Table 4:** Cases at different parameter setups.

Case	1	2	3	4	5	6	7	8	9
**max depth (Decision Trees)**	100	50	20	10	5	-	-	-	-
**Number of estimators (Random Forests)**	-	-	-	-	-	100	50	10	5

## 5 Evaluations on machine learning methods

Based on the above parametric studies, the results using the six machine learning algorithms with the corresponding optimal parameters in Table 5 were compared with each other. They were systematically evaluated from four aspects: prediction accuracy, computational time, feature sensitivity, and robustness on the failure of some hole port.

**Table 5:** Optimal parameters of the machine learning algorithms.

Abbreviation	Algorithms	Parameter
**KNN**	K-nearest neighbors	n_neighbors = 15, weights = ’distance’
**Poly**	Polynomial	5th
**MLP**	Multi-layer Perceptron	hidden_layer_sizes = (50,30),activation = ’relu’
**SVM**	Support Vector Machines	kernel = ’rbf’,C = 1e3
**DTs**	Decision Trees	max_depth = 50
**RFs**	Random Forests	n_estimators = 50

### 5.1 Prediction accuracy and computational time

**Fig 11:**
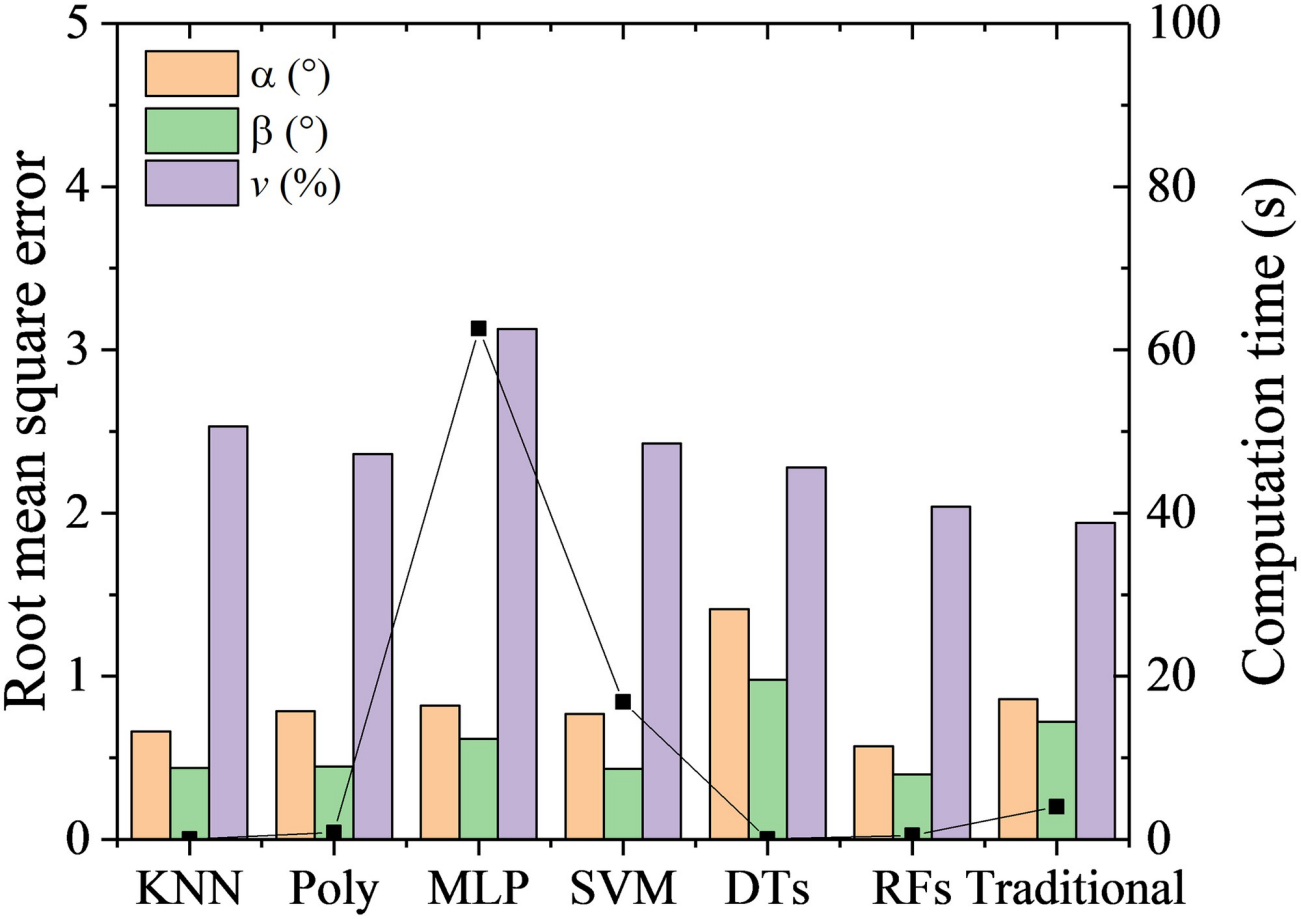
RMSEs and computation time with the six algorithms.

RMSEs and computation time with the six machine learning algorithms and the traditional method are shown in Fig 11. The prediction accuracy with the traditional method has RMSEs of 0.86° for *α*, 0.72° for *β* and 0.97% of *v*, which can be found in Wu’s previous work [25]. By comparison, the RFs has the highest prediction accuracy for both the flow angles and velocity. The RMSEs of calibration with RFs are 0.57° for *α*, 0.40° for *β* and 1.02% of *v*. The DTs has the lowest prediction accuracy for the flow angles, with RMSEs of 1.41° for *α*, and 0.98° for *β*. It is worth noting that RMSE of *β* are lower than *α* for all the algorithms. This is due to there are more pressure ports directly relating to *β* than *α*. For example, *β* are mainly dominated by pressure values of port 2, 3, 5 6 and 7, while *α* are mainly dominated by pressure values of port 1, 4 and 7, as shown in Fig 2. Therefore, for each algorithm, the more data relating to the feature are, the higher the prediction accuracy is.

Among the six algorithms, only MLP and SVM are time-consuming, which take 63s and 17s respectively. The other algorithms have high efficiency with computation time less than 1s, while the traditional method takes about 4s.

**Fig 12:**
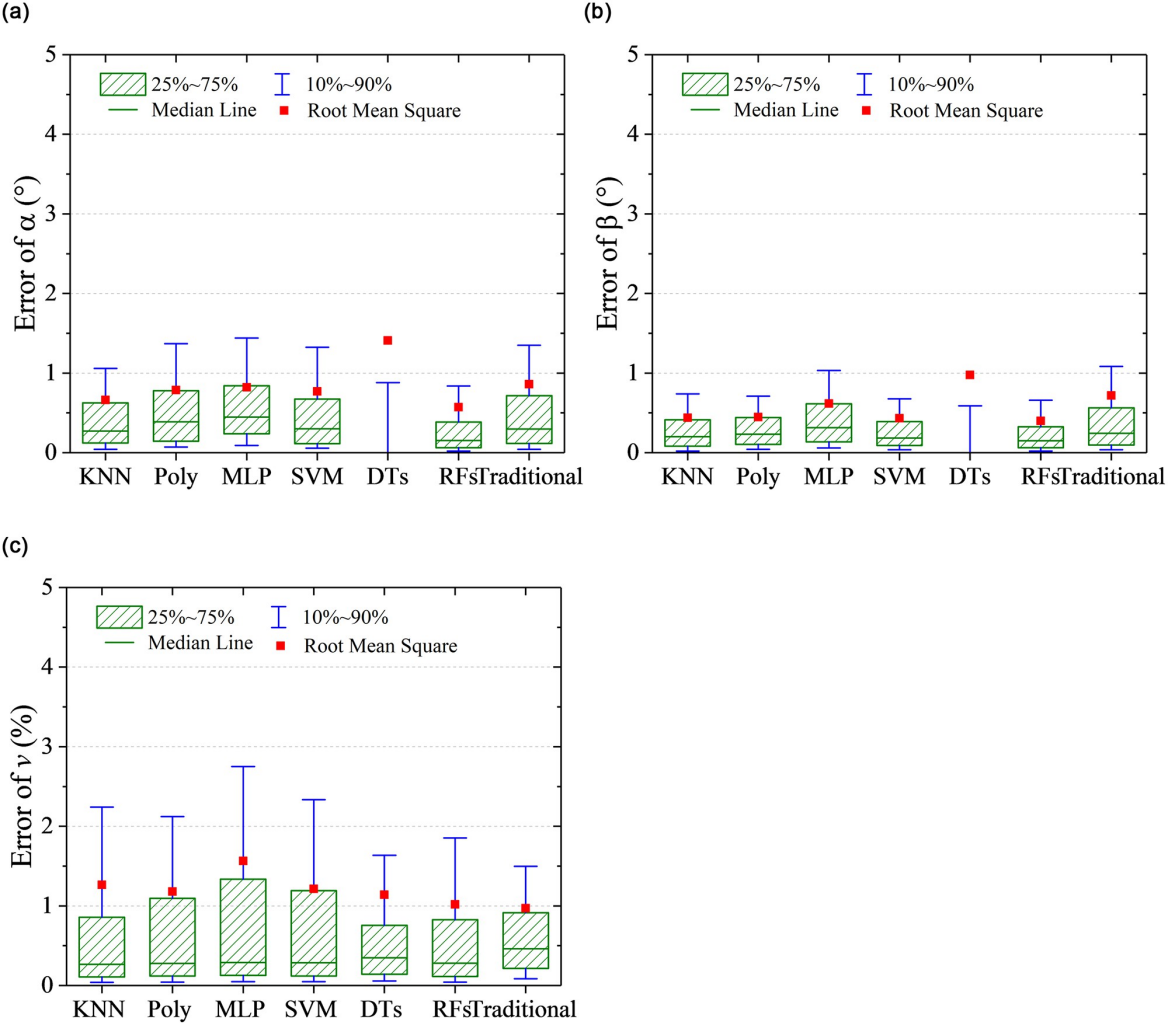
Quantile statistics on the prediction errors. (a) angle of attack, *α*, (b) sideslip angle, *β*, (c) flow velocity, *v*.

RMSE represents a global prediction error, which can not show the distribution information of the prediction error. Quantile statistic is another form of probability statistic, which provides the probability information of the prediction error less than some value. In this work, quantile statistics were conducted on the absolute value of the prediction errors to indicate the extent of deviation from the true value. As shown in Fig 12, five quantile points including 10%, 25%, 50%, 75%, and 90% are focused. It can be observed that the results of the DTs algorithm are special, of which the 75% quantile points of prediction errors on flow angles *α* and *β* are 0°. However, RMSEs of the predicted flow angles are the largest, which indicates that the DTs algorithm is easy to cause the over fitting. For the prediction on flow velocity, it is noticeable that KNN algorithm was used based on the predicted results of flow angles, which is illustrated in Fig 5. Hence, the quantile distribution of prediction error on flow velocity is different from it of prediction error on flow angles for the DTs algorithm. Overall, except for DTs algorithm, RMSEs are close to 75% quantile points, and the other quantile points show the same character with RMSEs for different algorithm. The RFs has the best prediction performance, of which 90% quantile of prediction errors on *α*, *β* and *v* are less than 0.8° and 0.6° and 2%. The prediction accuracy is acceptable for the flow measurement in wind tunnel or field tests.

**Fig 13:**
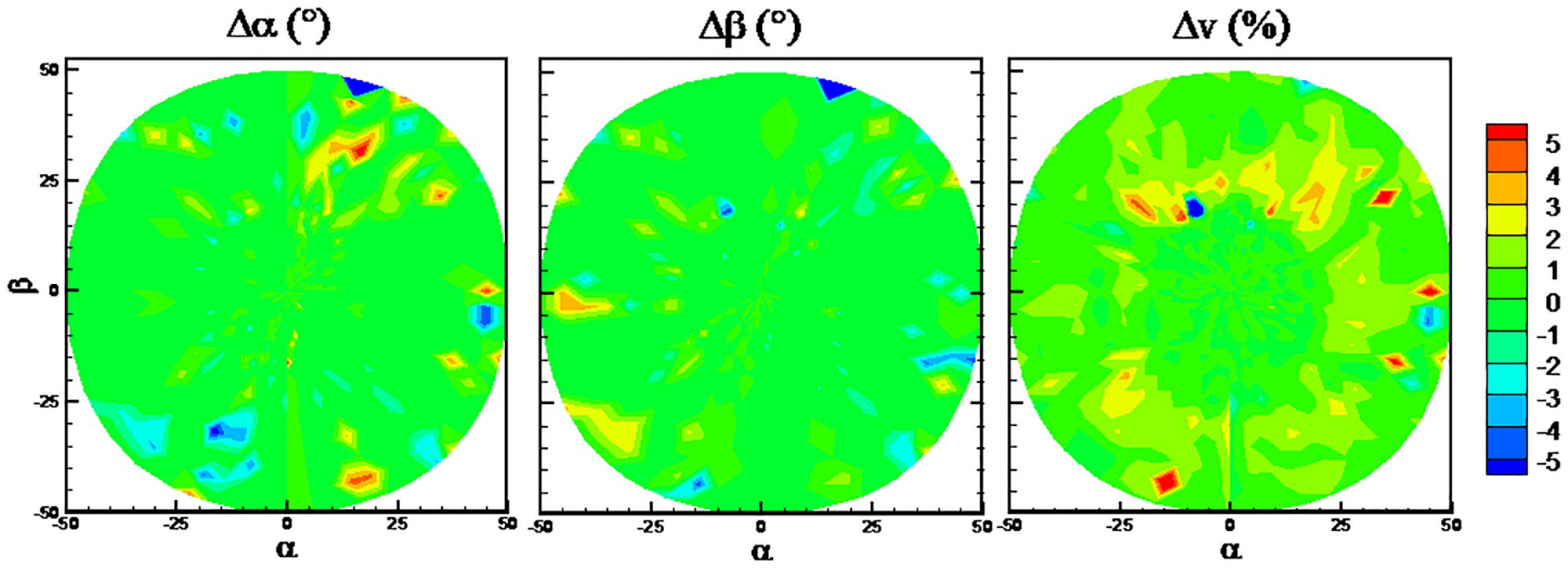
Error distributions with DTs algorithm.

**Fig 14:**
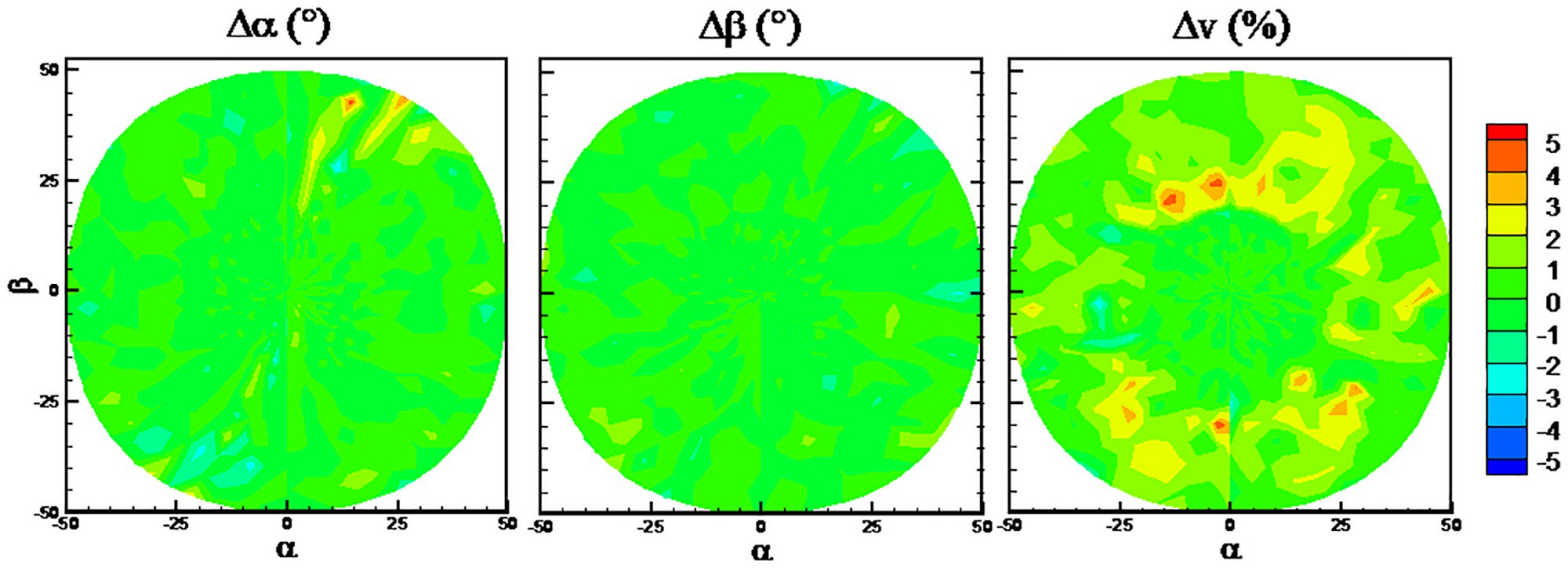
Error distributions with RFs algorithm.

**Fig 15:**
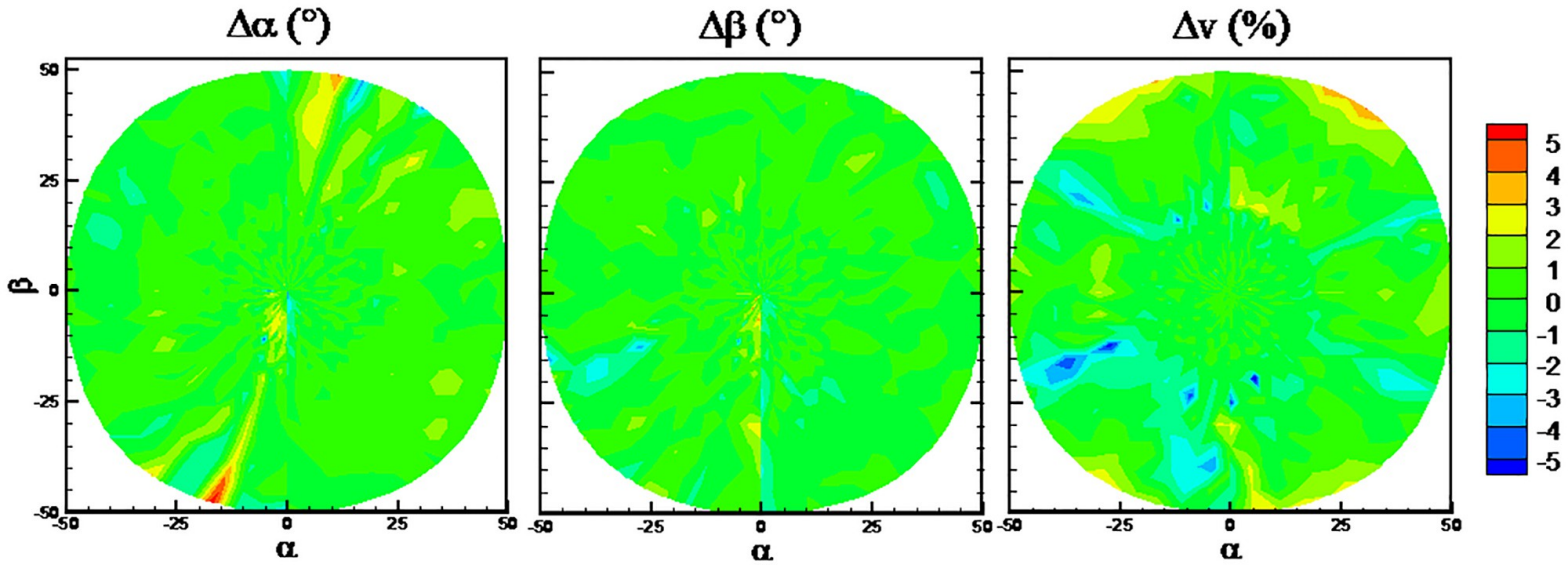
Error distributions with the traditional method.

The DTs algorithm has maximum RMSEs on the predicted flow angles, while the RFs algorithm has minimum RMSEs. The detailed error distributions of these two algorithms are shown in Figs 13 and 14, respectively. The error distributions of the traditional method are shown in Fig 15 as a comparison group. By comparison, there are more randomly distributed data points with prediction errors larger than 5° for the DTs algorithm, which results in the larger RMSEs.

### 5.2 Feature sensitivity

In the above studies, feature in Eq (2) based on the aerodynamic knowledge was chosen, and the acceptable predicted accuracy was obtained. However, as a mathematical technology, machine learning can still work without the physical knowledge. Therefore, the effects of feature extraction on the prediction accuracy are focused on in this section to investigate the feature sensitivity.

#### 5.2.1. Machine learning with feature in Eq (3).

**Fig 16:**
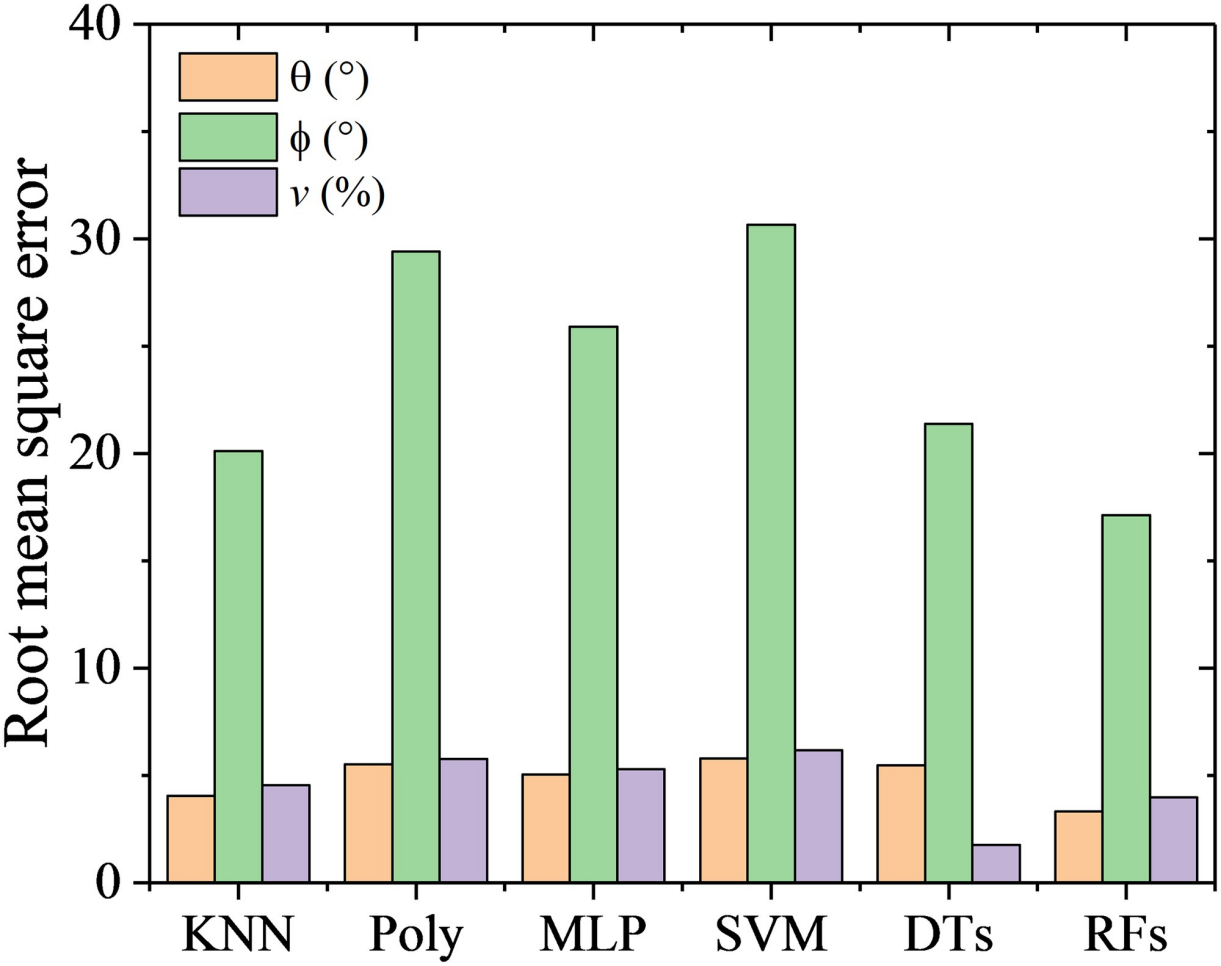
RMSEs using feature in Eq (3).

**Fig 17:**
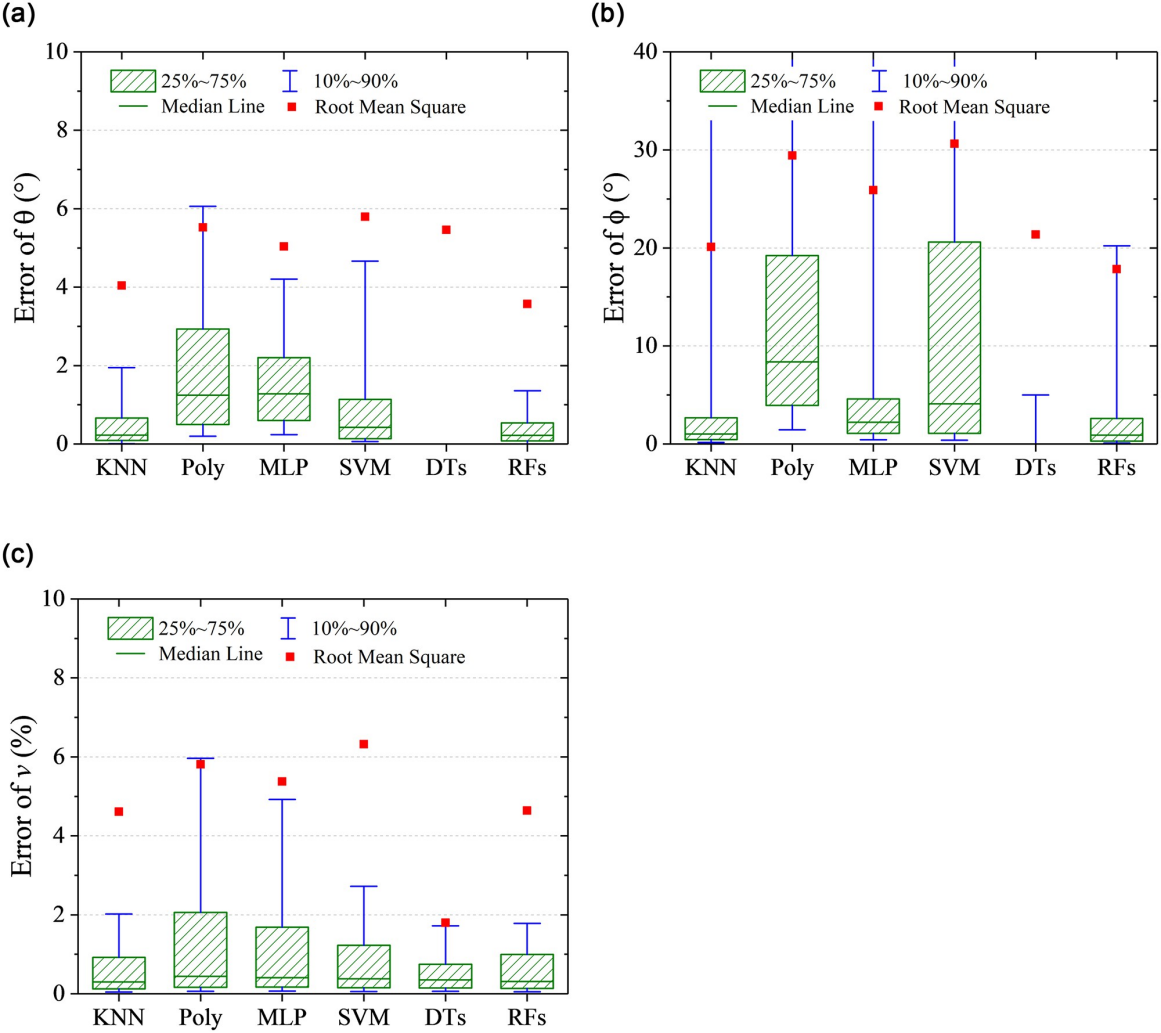
Quantile statistics on the prediction errors. (a) pitching angle, *θ*, (b) rolling angle, *ϕ*, (c) flow velocity, *v*.

 Based on the feature in Eq (3), machine learning methods are used to fit the function between flow angles (*θ*, *ϕ*) and the normalized pressure coefficients. The RMSEs of flow angles (*θ*, *ϕ*) and velocity are shown in Fig 16. Obviously, a large and unacceptable prediction error on rolling angle is obtained for all the machine learning algorithms. It is shown from quantile statistics in Fig 17 the RMSEs of pitching angle and flow velocity are close to or even larger than 90% quantile points, which indicates that the errors at 10% data points are huge and abnormal. In comparisons, the SVM algorithm has maximum RMSEs on the predicted flow angles, while the RFs algorithm has minimum RMSEs.

**Fig 18:**
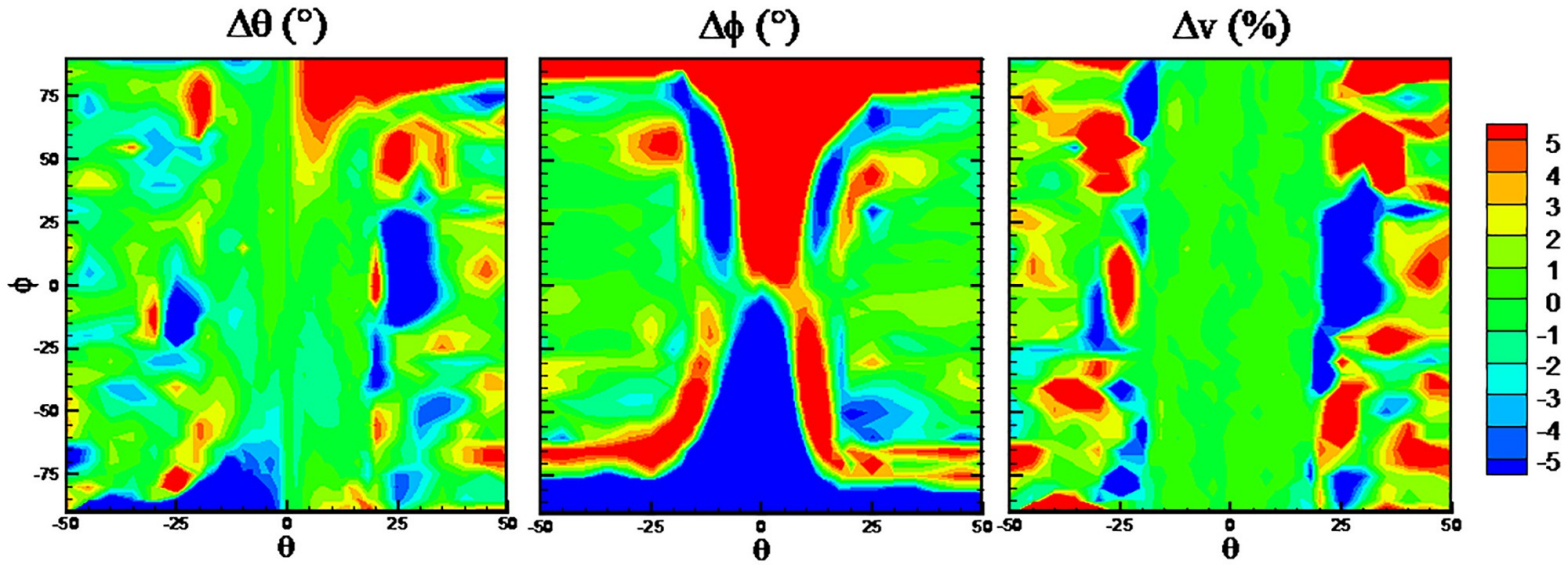
Error distributions with SVM algorithm.

**Fig 19:**
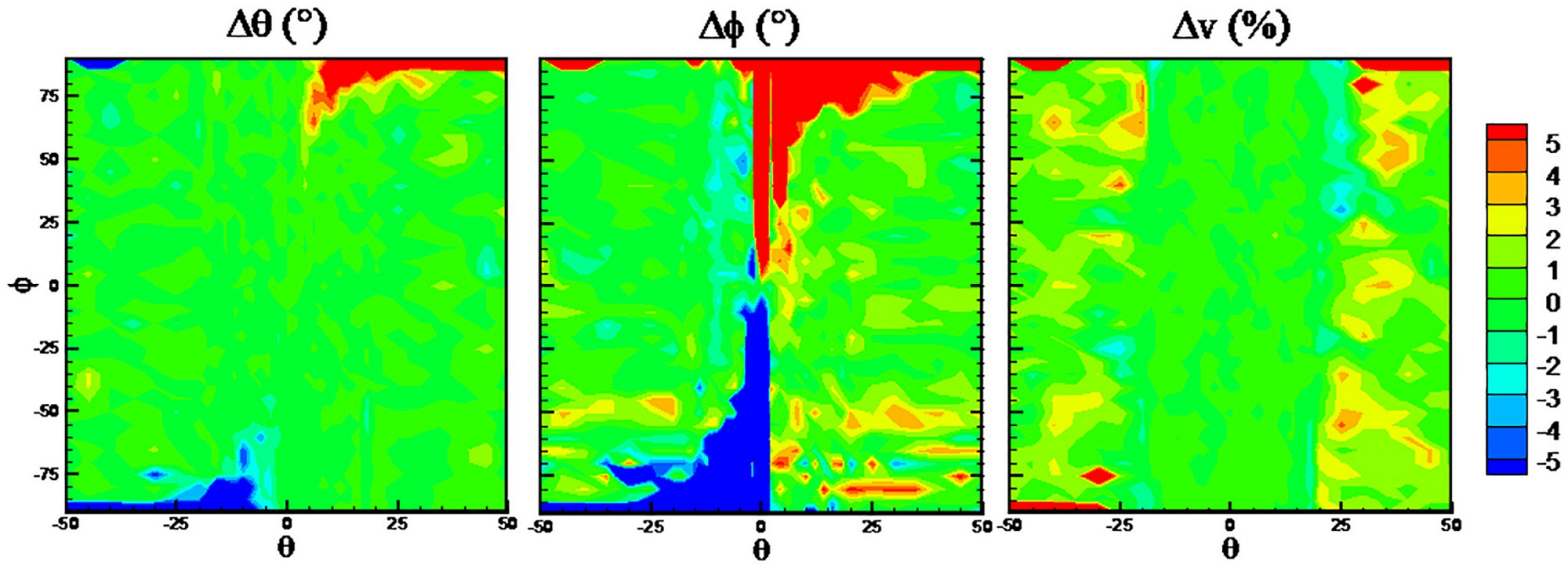
Error distributions with RFs algorithm.

**Fig 20:**
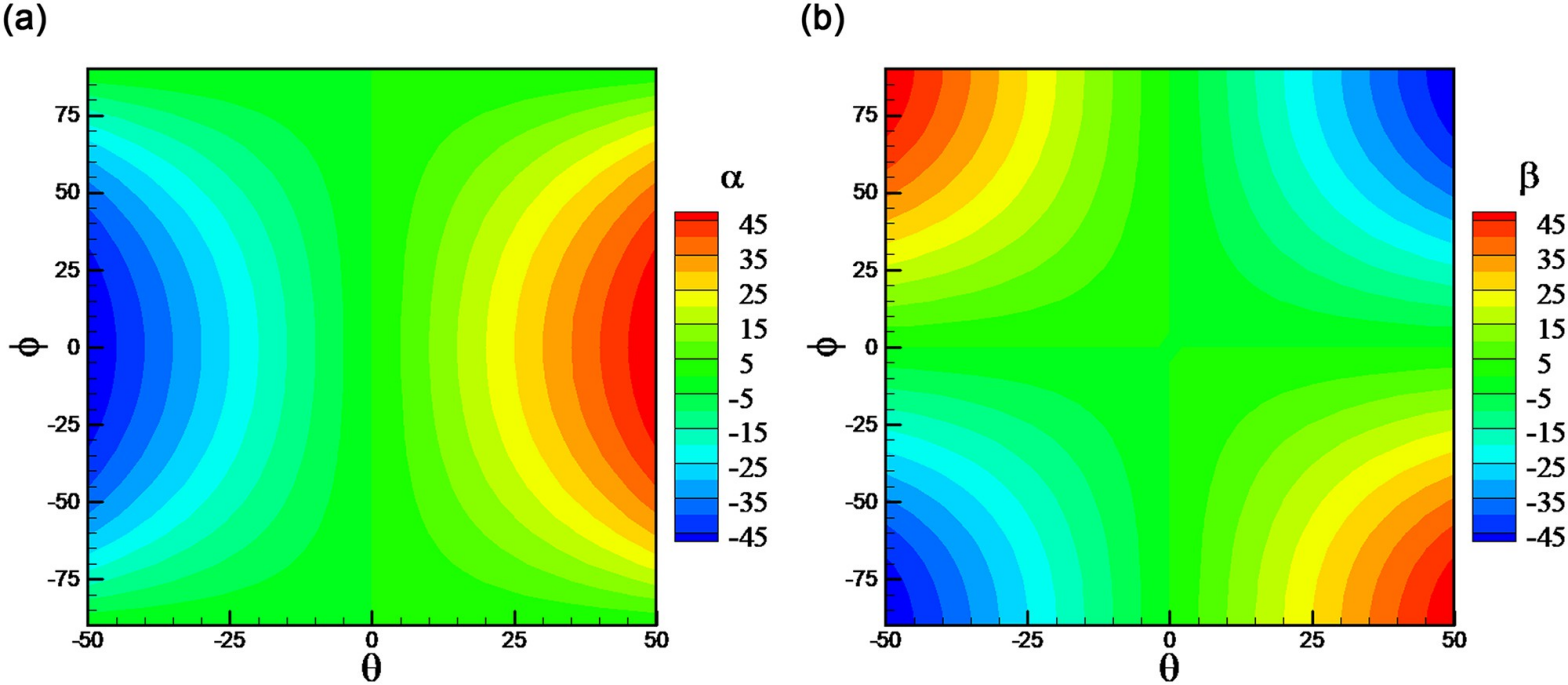
The relationships between *θ* ~ *ϕ* and *α* ~ *β*. (a) *α* ~ *θ* (b) *β* ~ *θ*, *ϕ*.

Figs 18 and 19 present the error distributions in *θ* ~*ϕ* coordinates. Firstly, there are more points with larger errors when using SVM algorithm. Secondly, larger errors of pitching angle appear at the high rolling angle points, while larger errors of rolling angle appear at the points with pitching angle of 0°. This is due to that port pressure values are directly sensitive to *α* and *β*, rather than *θ* and *ϕ*. The relationships between *θ* ~ *ϕ* and *α* ~ *β* are shown in Fig 20. It can be observed at the high rolling angle, for example 90° or -90°, *α* will be irrelative with *θ*, which means different pressure values will be corresponding to one pitching angle. This leads to larger error of predicted pitching angle at the high rolling angle points. Similarly, at pitching angle of 0°, *α* and *β* will be both irrelative with *ϕ*, which leads to larger error of predicted rolling angle, as shown in Figs 18 and 19.

#### 5.2.2. Machine learning with feature in Eq (4).

 The basic principle of multi-hole probe calibration is to find out the relationship between flow parameters and port pressure values. The function in Eq (4) is the initial and direct relation without any preprocessing on the pressure data. Based on the feature in Eq (4), machine learning methods are used to fit the function between flow parameters (*α*, *β*, *dp*) and the pressure coefficients.

**Fig 21:**
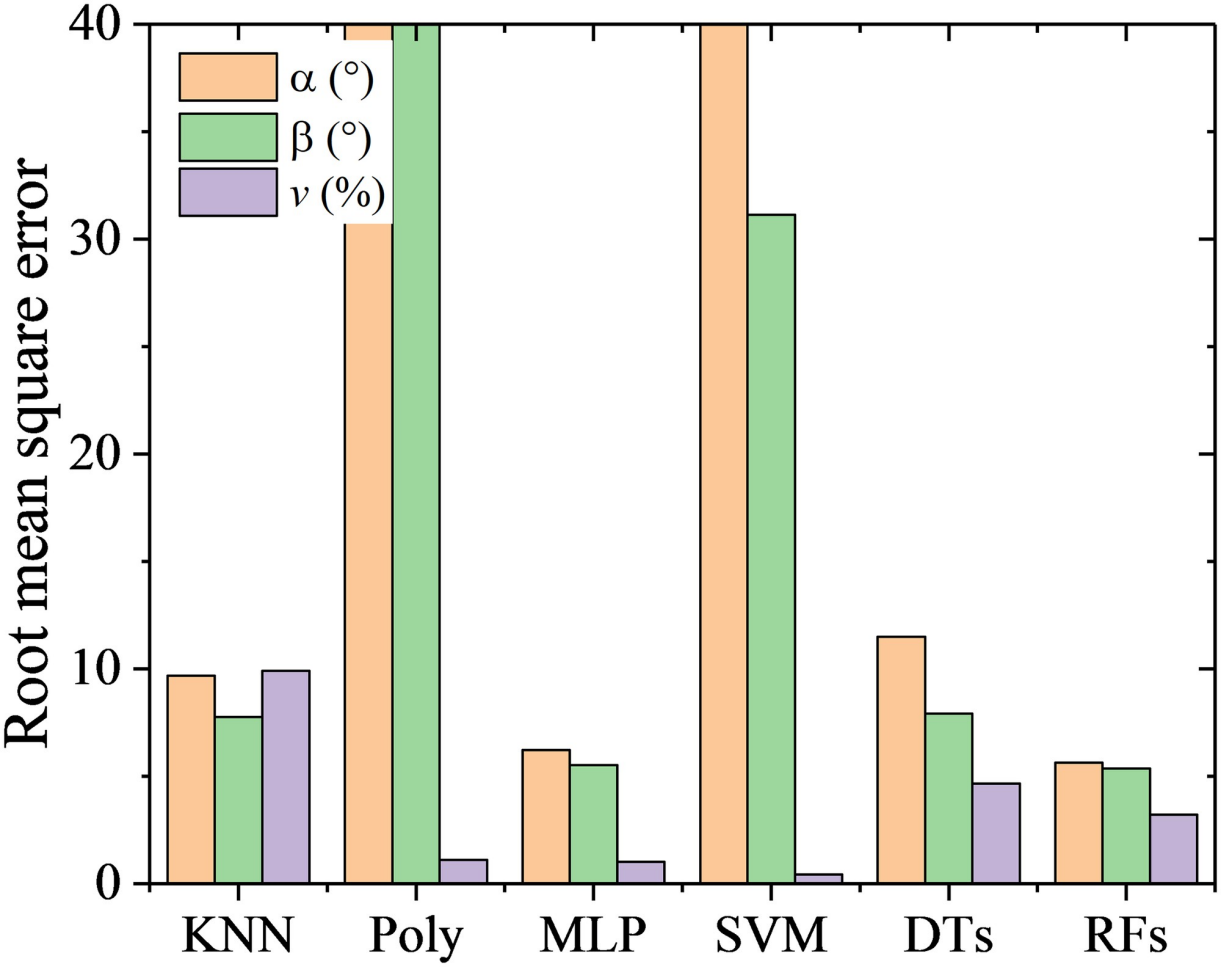
RMSEs using feature in Eq (4).

The RMSEs of flow angles (*α*, *β*) and velocity *v* are shown in Fig 21. Obviously, the predicted accuracy of flow velocity is higher than it of the flow angles for most of the machine learning method. This is due to the port pressure values are proportional to the square of flow velocity, while they vary with flow angles in a linear manner approximately. When the flow angles and velocity are predicted simultaneously, the flow velocity will be a dominant feature and weaken the effects of flow angles on the pressure values.

**Fig 22:**
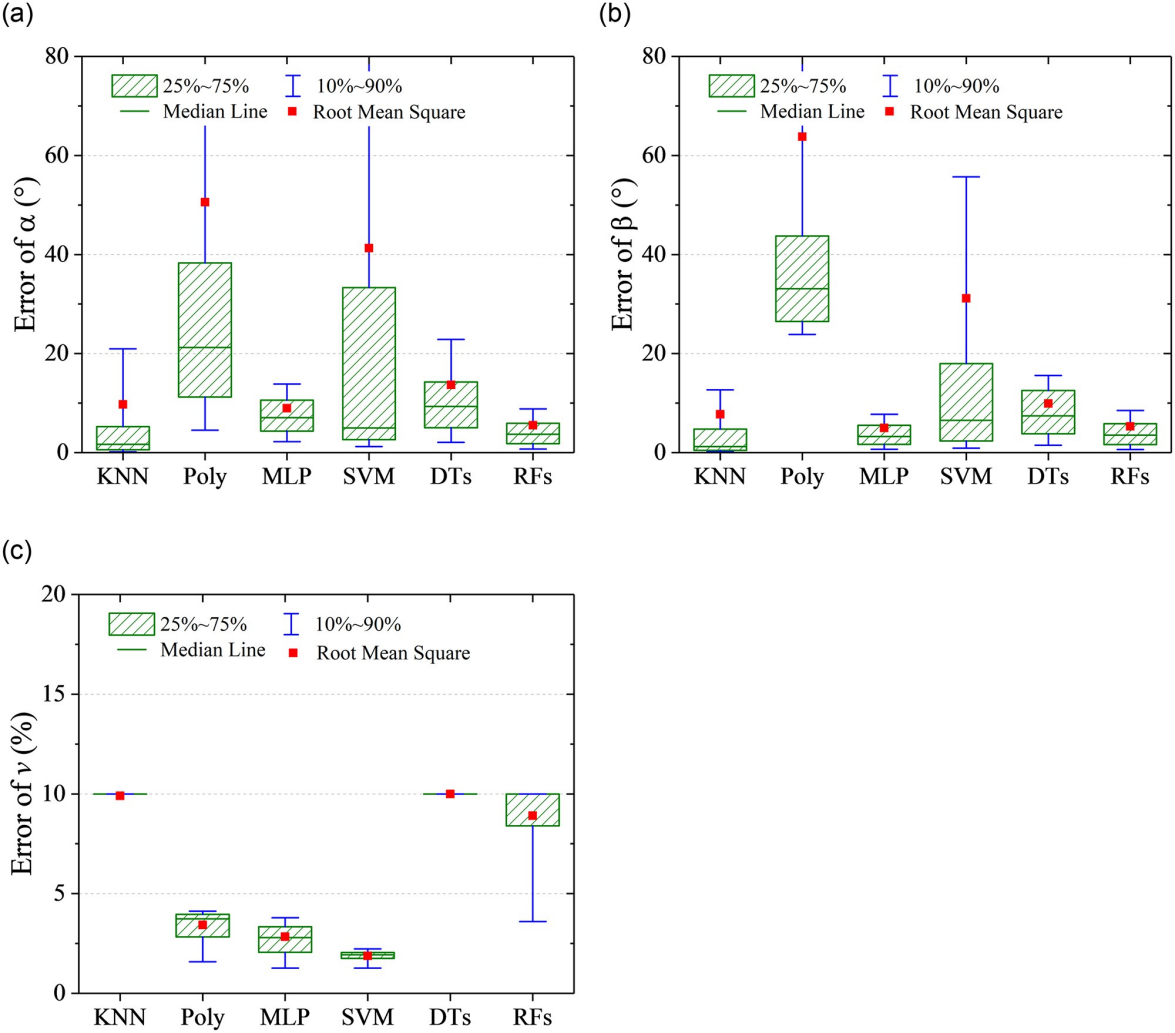
Quantile statistics on the prediction errors. (a) angle of attack, *α* (b) sideslip angle, *β* (c) flow velocity, *v*.

It can be observed from the quantile statistics in Fig 22 that Poly, MLP and SVM algorithms have significant advantages in predicting the flow velocity, but they are weak in predicting the flow angles. This indicates that Poly, MLP and SVM algorithms are suitable to find out the main feature, while KNN, DTs and RFs algorithms are suitable to solve the prediction problem with high density training samples. In the training dataset, only two flow velocities, 10 m/s and 30 m/s, are included, while high density flow angles are included.

**Fig 23:**
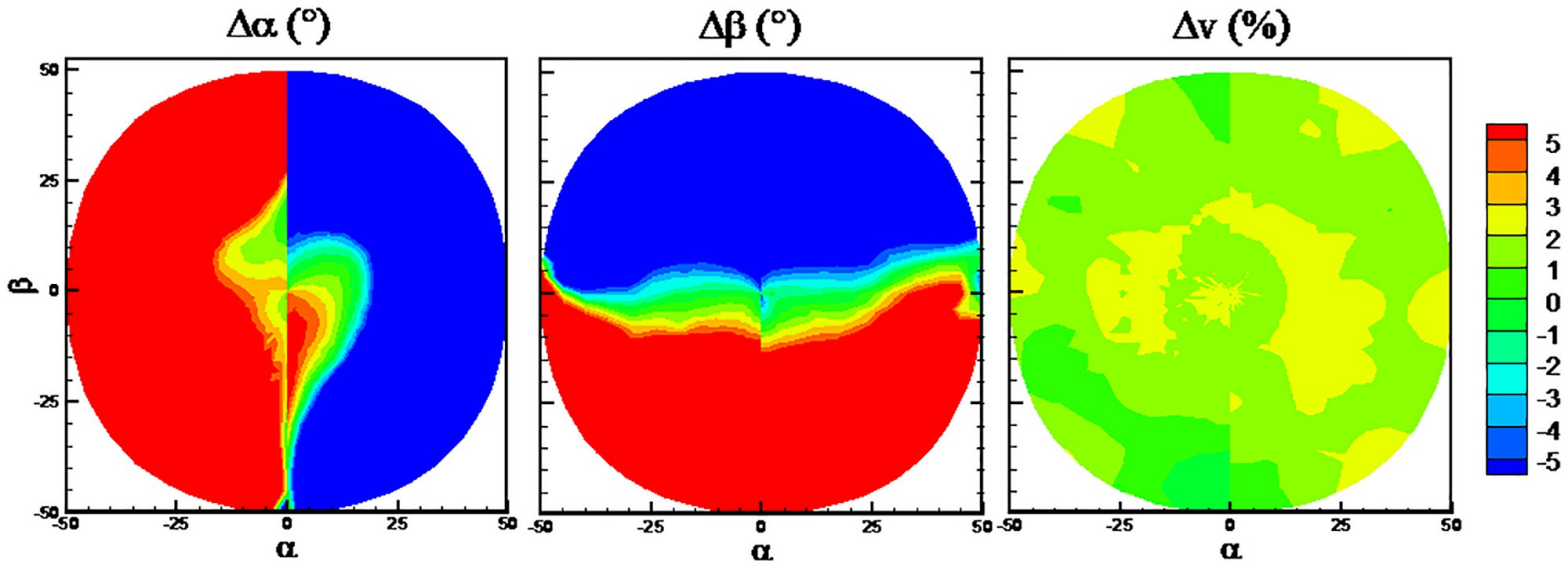
Error distributions with SVM algorithm.

**Fig 24:**
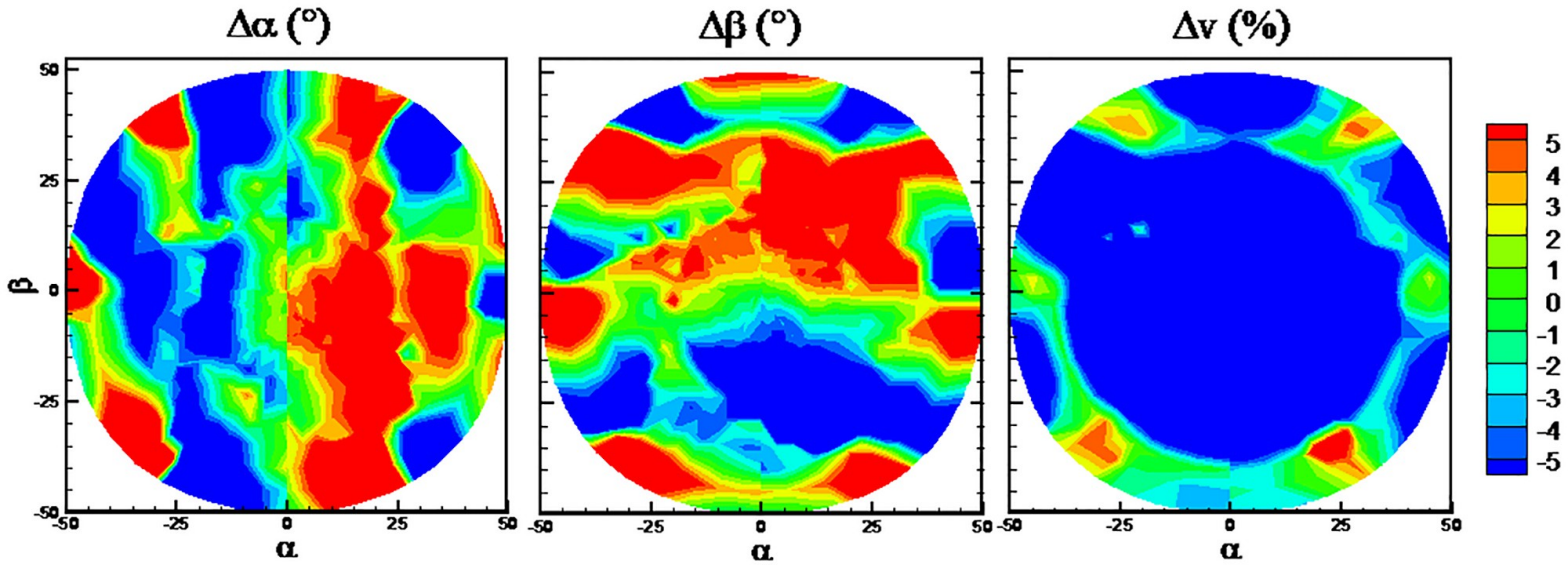
Error distributions with RFs algorithm.

Figs 23 and 24 present the error distributions with SVM and RFs algorithm. Only the predicted flow velocity by SVM is satisfactory.

In general, the predicting accuracy is very sensitive to the features from above studies. Without appropriate features, the machine learning methods may be invalid. Hence, feature extraction plays an important role in the machine learning methods. In the practice process, the combination of physical knowledge and data mining is the best solution for high accuracy predictions.

### 5.3 Robustness on the failure of some hole port

In hostile environments, such as sandstorms in wind farm, some port may be malfunctioning. For a seven-hole probe that port number is defined in Fig 1, Port 4 is symmetric with Port 1, and Port 2 is symmetric with Port 3, 5, and 6. Therefore, the cases that Port 1, 2 and 7 is invalid were studied, respectively.

**Fig 25:**
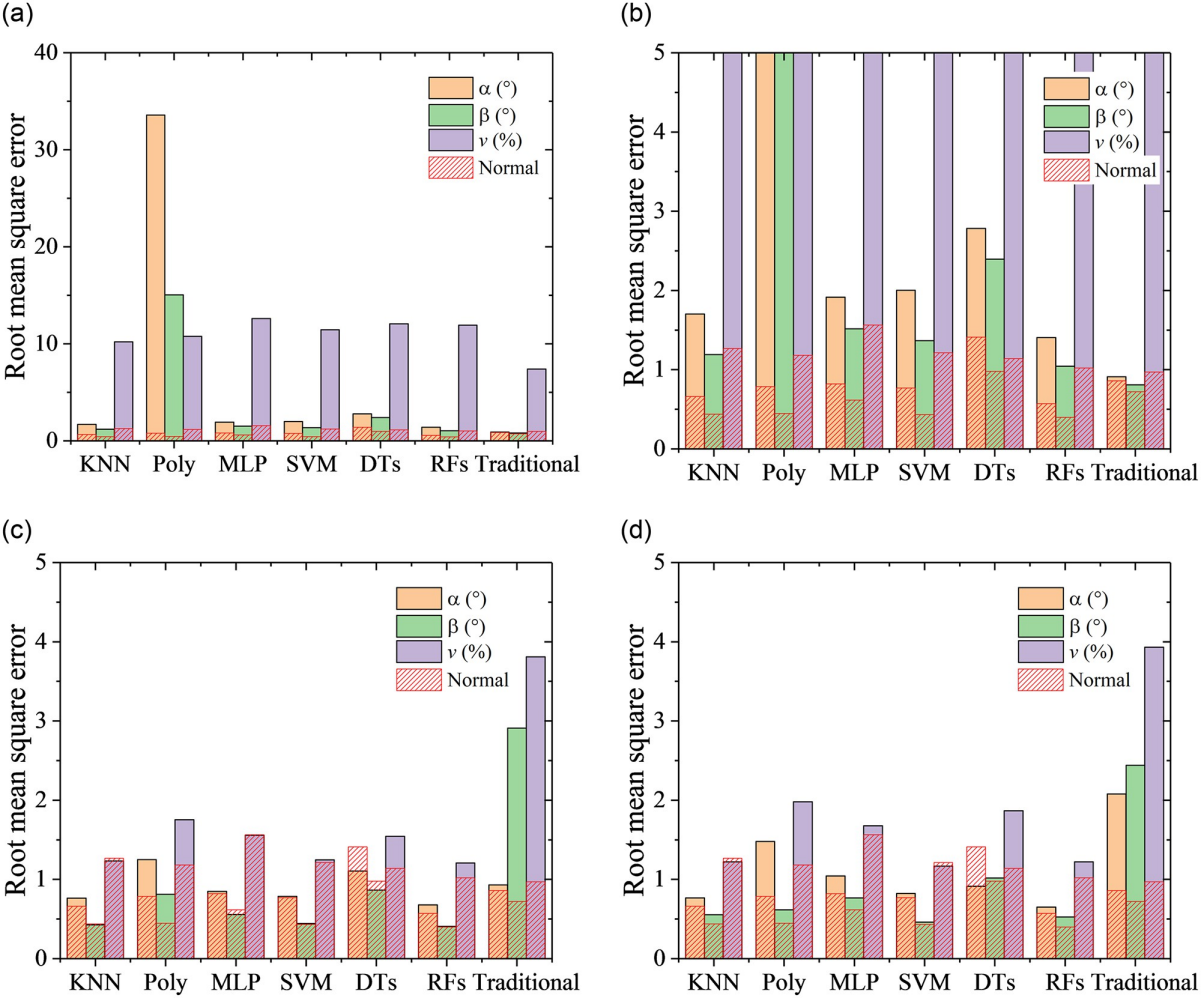
RMSEs with the six algorithms. a) port 7 is invalid (zoom out), b) port 7 is invalid, c) port 1 is invalid, d) port 2 is invalid.

**Fig 26:**
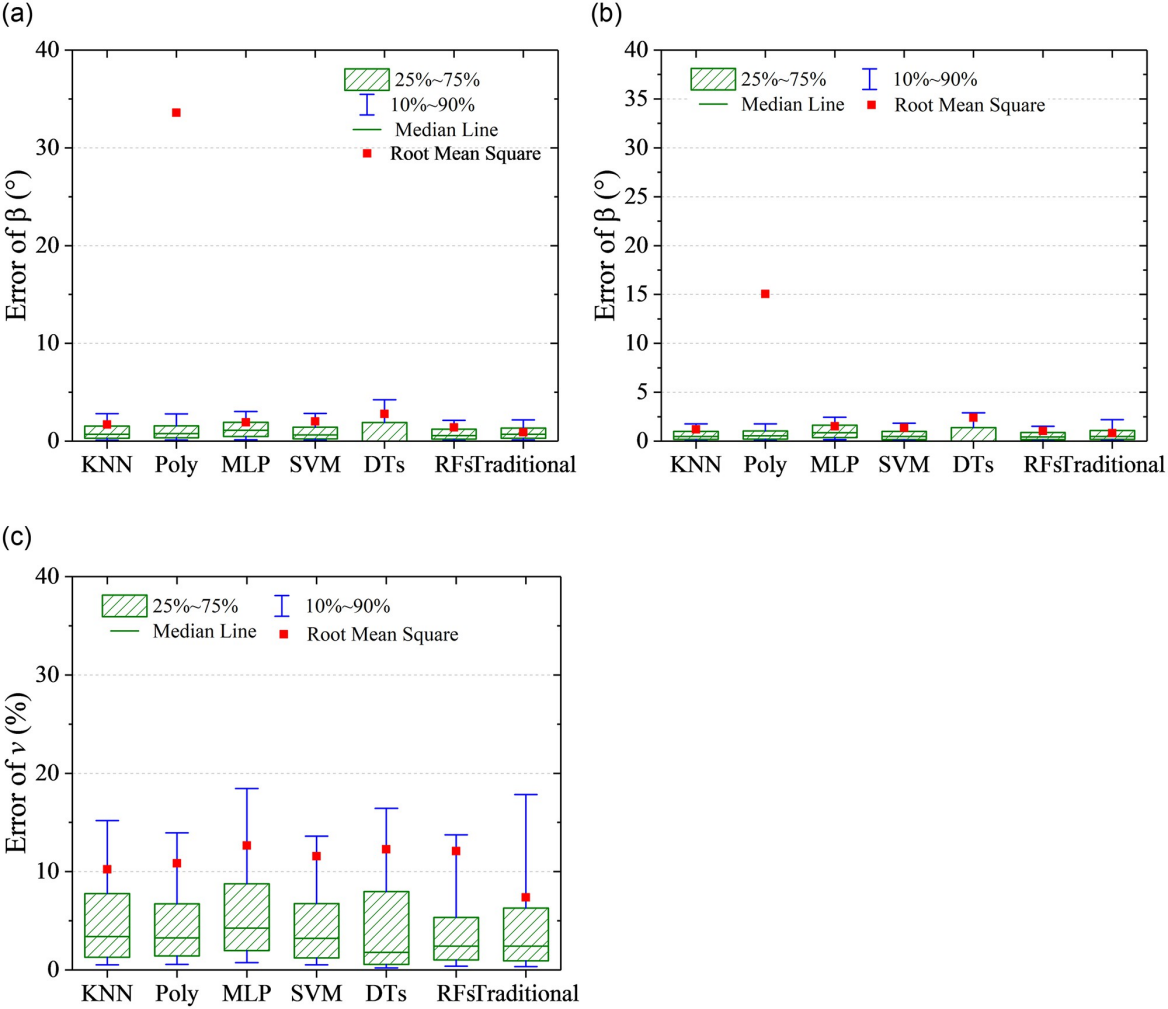
Quantile statistics on the prediction errors when port 7 is invalid. (a) angle of attack, *α*, (b) sideslip angle, *β*, (c) flow velocity, *v*.

**Fig 27:**
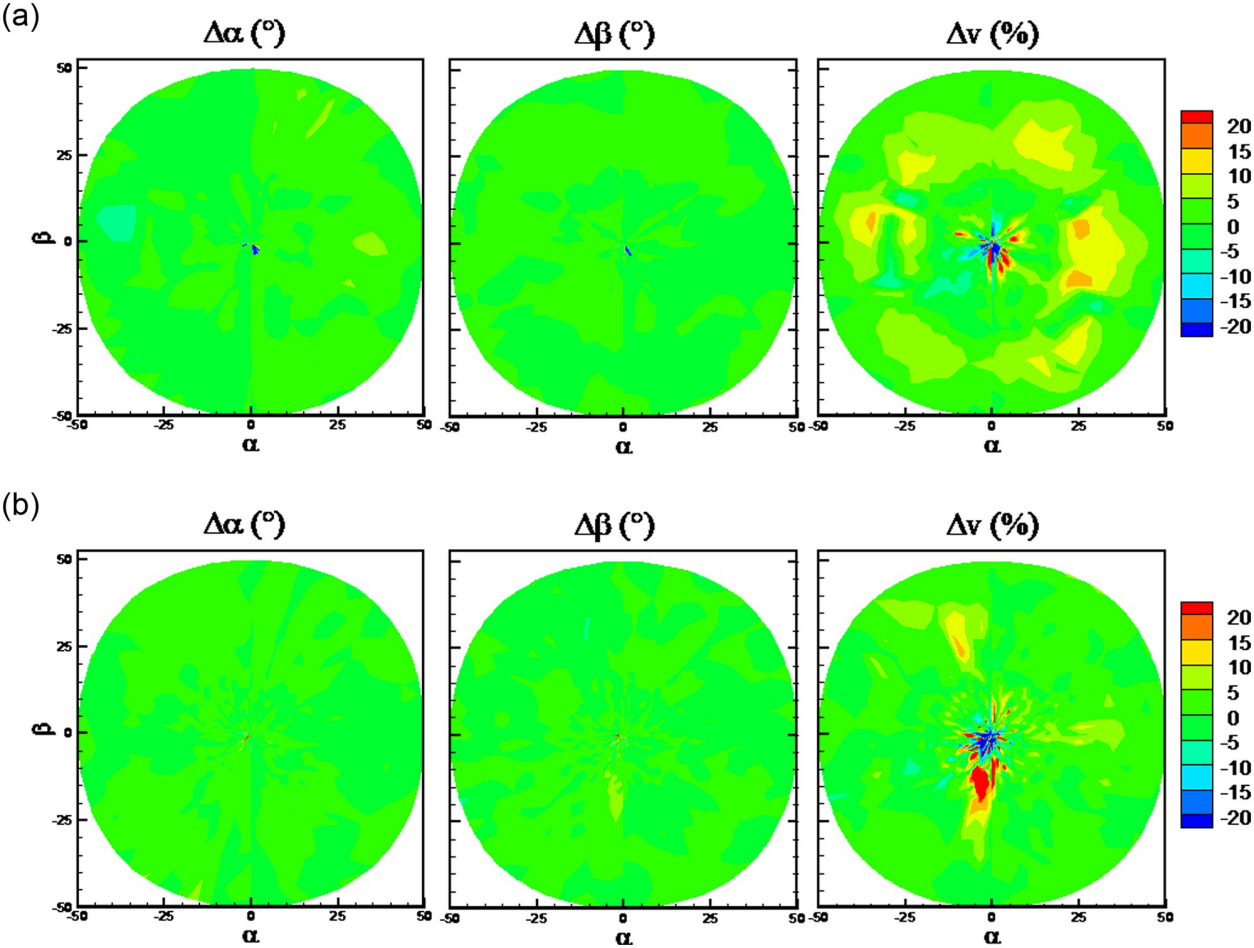
Error distributions when port 7 is invalid. a) Poly algorithm, b) traditional method.

The RMSEs with the six algorithms and the traditional method are shown in Fig 25. It can be observed from Fig 25(a) and 25(b) that when port 7 is invalid, the prediction accuracy of flow velocity becomes worsen markedly for all the algorithms in comparison with the normal case. In the normal case in Fig 11, the RMSEs of predicted flow velocity are close to 1%, while they are close to 10% when port 7 is invalid. By comparison, the traditional method has the highest predicted accuracy when port 7 is invalid. Meanwhile, the RMSEs of predicted flow angles are about two times compared to the normal case, except the Poly algorithm. Fig 26 presents the quantile statistics on the prediction errors when port 7 is invalid. It can be observed that RMSEs of flow angles are much higher than the 90% quantile for the Poly algorithm, which indicates there are some abnormal predicted results. It is shown from the error distributions in Fig 27 that compared with the traditional method, the predicted errors at the region of flow angles close to 0° are huge and abnormal. It is worth noting that the color-coded values are from -20 to 20 in Fig 27, which is different from the other error distribution figures.

It can be observed from Fig 25(c) and 25(d) that when port 1 or 2 is invalid, the prediction accuracy of flow velocity and angles are slightly affected except Poly and DTs algorithms. It is worth noting that the prediction accuracy of the traditional method deteriorates significantly when port 1 or 2 is invalid. This indicates that the machine learning method is more robust than the traditional method.

**Fig 28:**
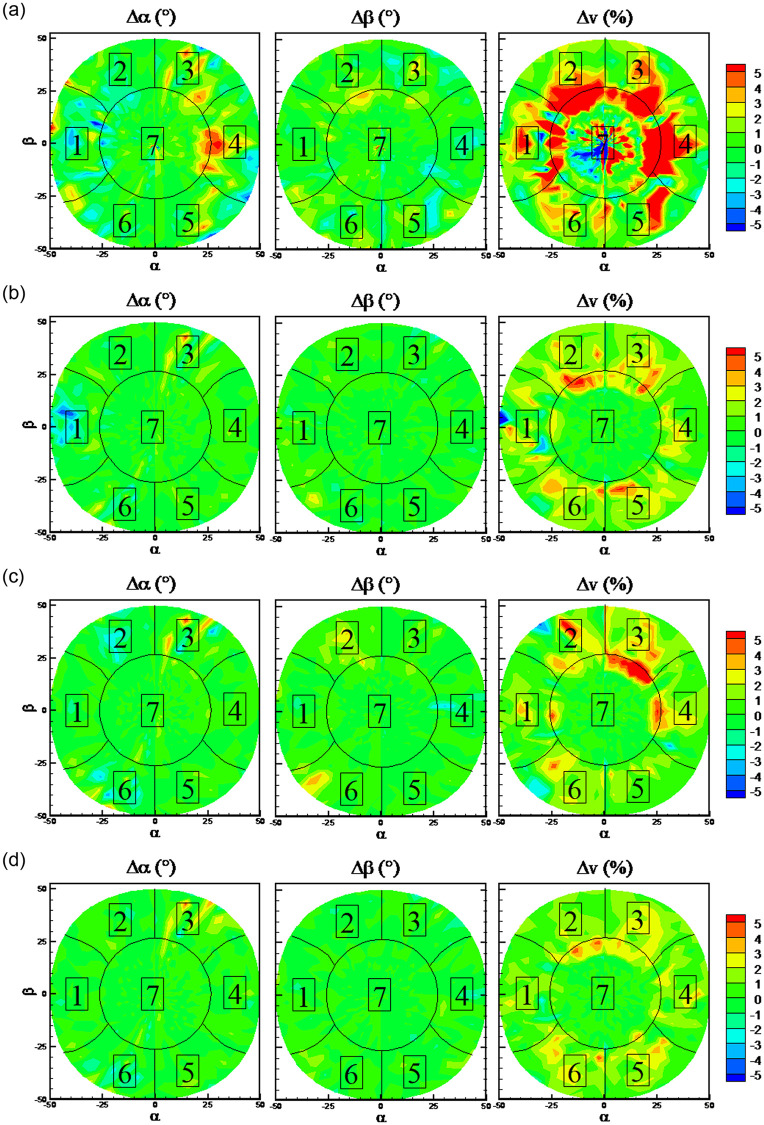
Error distributions with RFs algorithm. a) port 7 is invalid, b) port 1 is invalid, c) port 2 is invalid, d) normal case: all ports are valid.

**Fig 29:**
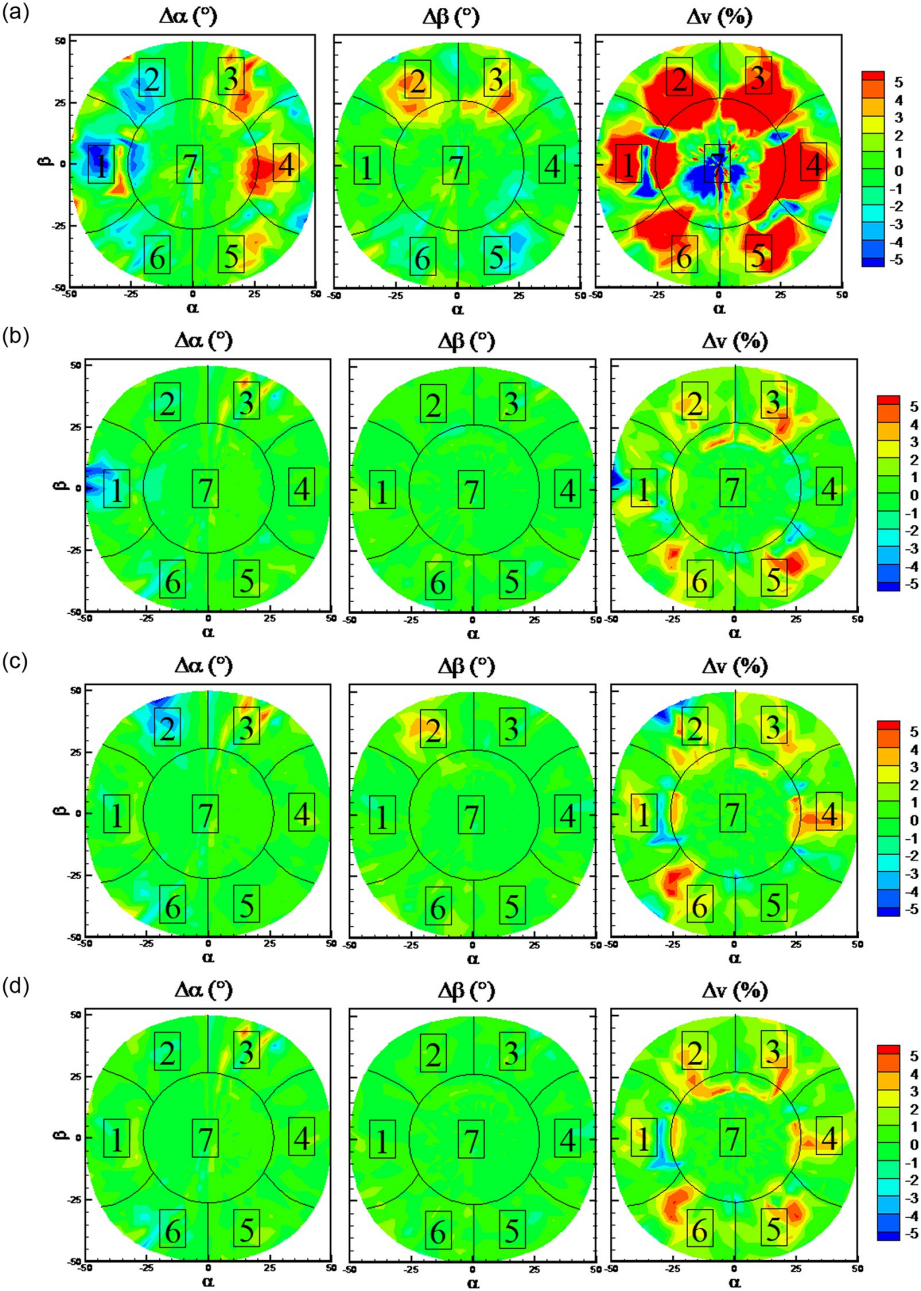
Error distributions with KNN algorithm. a) port 7 is invalid, b) port 1 is invalid, c) port 2 is invalid, d) normal case: all ports are valid.

It can be observed from Figs 11 and 25 that RFs and KNN algorithm presents the best predicted performance. The error distributions with RFs and KNN algorithms on the three cases when some port is invalid and all ports are valid are shown in Figs 28 and 29 to study the effects of the missing port data. Numbers from 1 to 7 in different sectors of Figs 28 and 29 represents that the pressure of this port number are maximum in this sector. Black lines in Figs 28 and 29 are the theoretical boundary of different sectors. When Port 7 is invalid, errors in all the sector are increased in comparison with the normal case. When Port 1 is invalid, errors of flow angles only in sector 1 are increased, and errors of flow velocity in all the sector are increased. When Port 2 is invalid, errors of flow angles only in sector 2 are increased, and errors of flow velocity in all the sector are increased. These results from Figs 25, 28 and 29 indicate that the missing of port 7 data will increase the predicted errors of all the flow parameters at all the sectors, while the missing of the other port data has slightly effects on the predicted errors of flow angles at the region except the corresponding sector. Overall, the prediction accuracy is acceptable for the probe with one invalid port.

### 5.4 Comprehensive evaluation on the machine learning methods

The above studies have discussed the prediction performance of the six machine learning algorithms in detail from four aspects: prediction accuracy, computational time, feature sensitivity, and robustness on the failure of some hole port. Based on the findings, a min-max normalization study was conducted to present a quantitative and comprehensive evaluation on the machine learning methods. The min-max normalization is defined as Eq (6), where *x* is the value of RMSEs or the computational time, min is 0, and max is the maximum value of all the RMSEs or the computational time.


$$C_{x}=1-\left(x-\text{min}\right)/\left(\text{max}-\text{min}\right)$$ (6)

**Fig 30:**
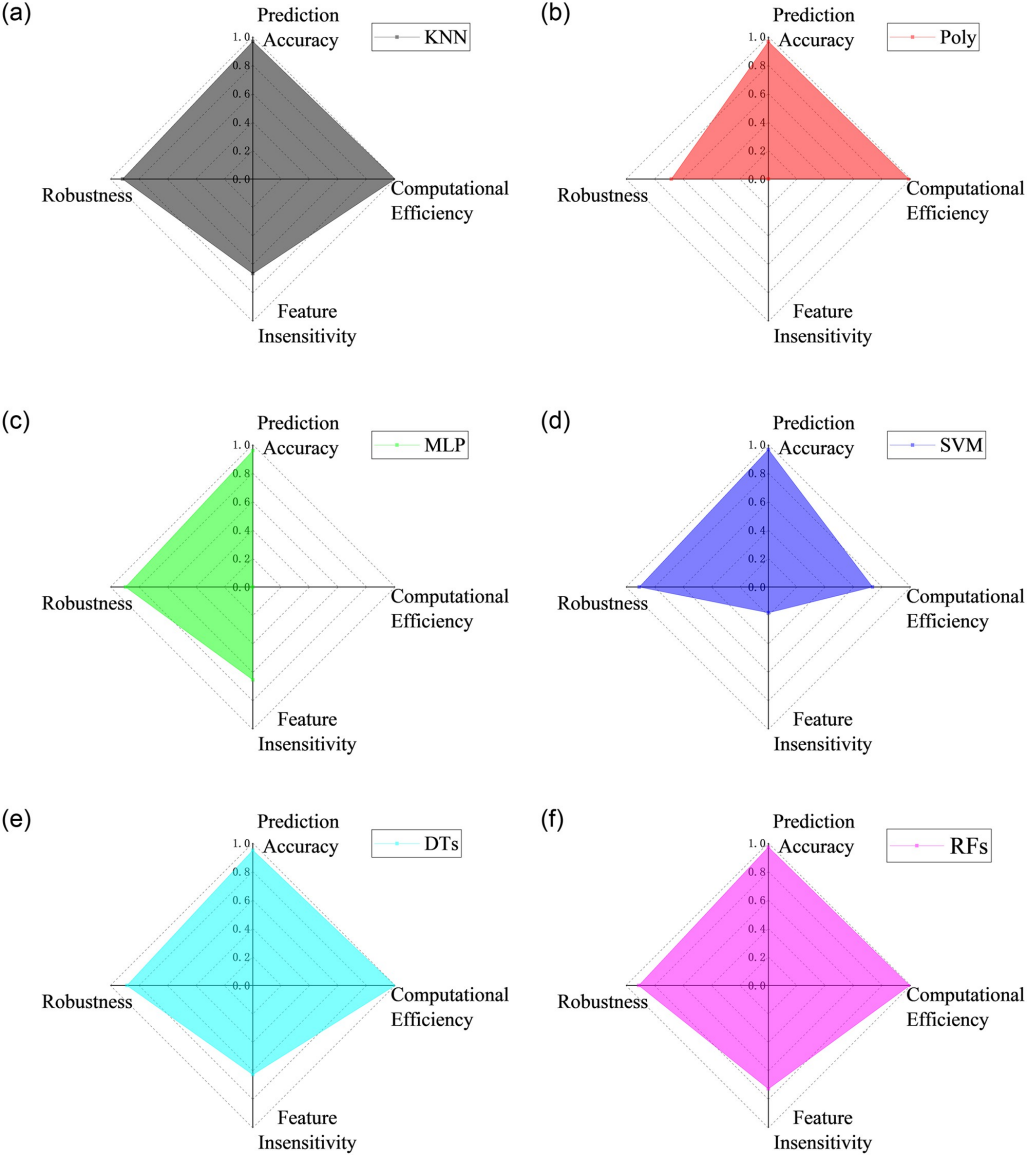
Comprehensive evaluation on the six machine learning algorithms.

Fig 30 presents the comprehensive prediction performance of the six algorithms. For the prediction accuracy, all the algorithm can obtain the satisfactory predicted results when choosing the appropriate parameters and features. In contrast, DTs typically exhibit high variance and tend to overfit. For the computational efficiency, only MLP and SVM are time-consuming, which take 63s and 17s respectively. The other algorithms have high efficiency with computation time less than 1s. The machine learning methods have the great advantage in the computational efficiency in comparison with the in-house traditional method. The detailed description can be found in the publication [25]. For the feature insensitive, all the algorithms are very sensitive to the features. Using the features based on the physical knowledge can obtain a high accuracy predicted results. In contrast, Poly, MLP and SVM algorithms are suitable to find out the most important feature without physical knowledge, while KNN, DTs and RFs algorithms are suitable to solve the prediction problem with high density training samples. For the robustness on the failure of some hole port, the missing of central port data will reduce the prediction accuracy of flow velocity from 1% to about 10% with all the algorithms, and the prediction accuracy of flow angles can not be accepted only with Poly algorithm. The missing of surrounding port data has slight effects on the prediction accuracy, and all the algorithms can obtain a high and acceptable prediction accuracy.

Overall, RFs and KNN algorithms have the better comprehensive prediction performance. Meanwhile, they have the great advantages in the computational efficiency and the convenience of writing code, compared with the in-house traditional algorithm that takes about 100 times as long.

## 6 Application in measuring angle of attack of a wind turbine blade in field

**Fig 31:**
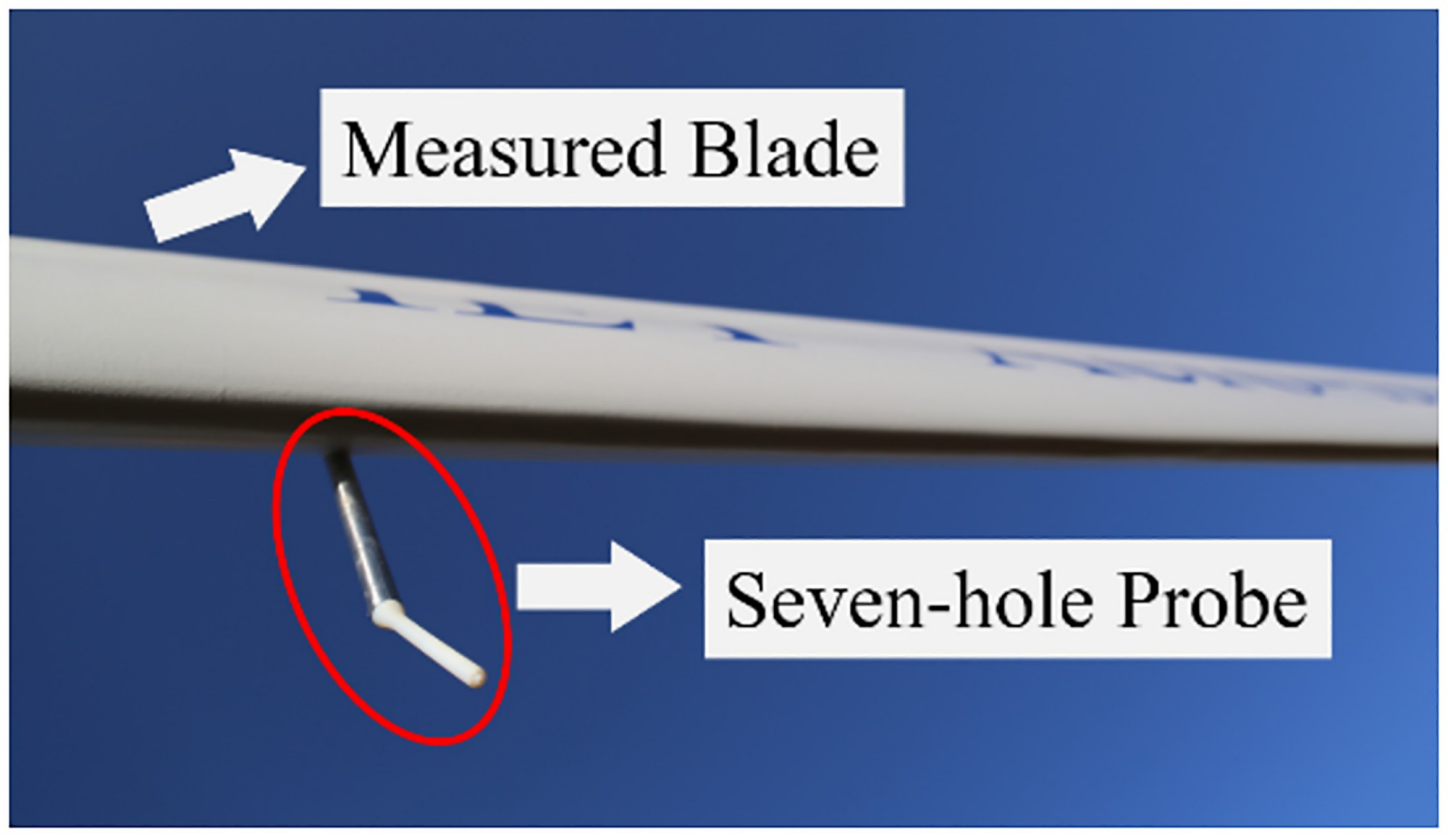
Photo of the probe mounted on the blade.

**Fig 32:**
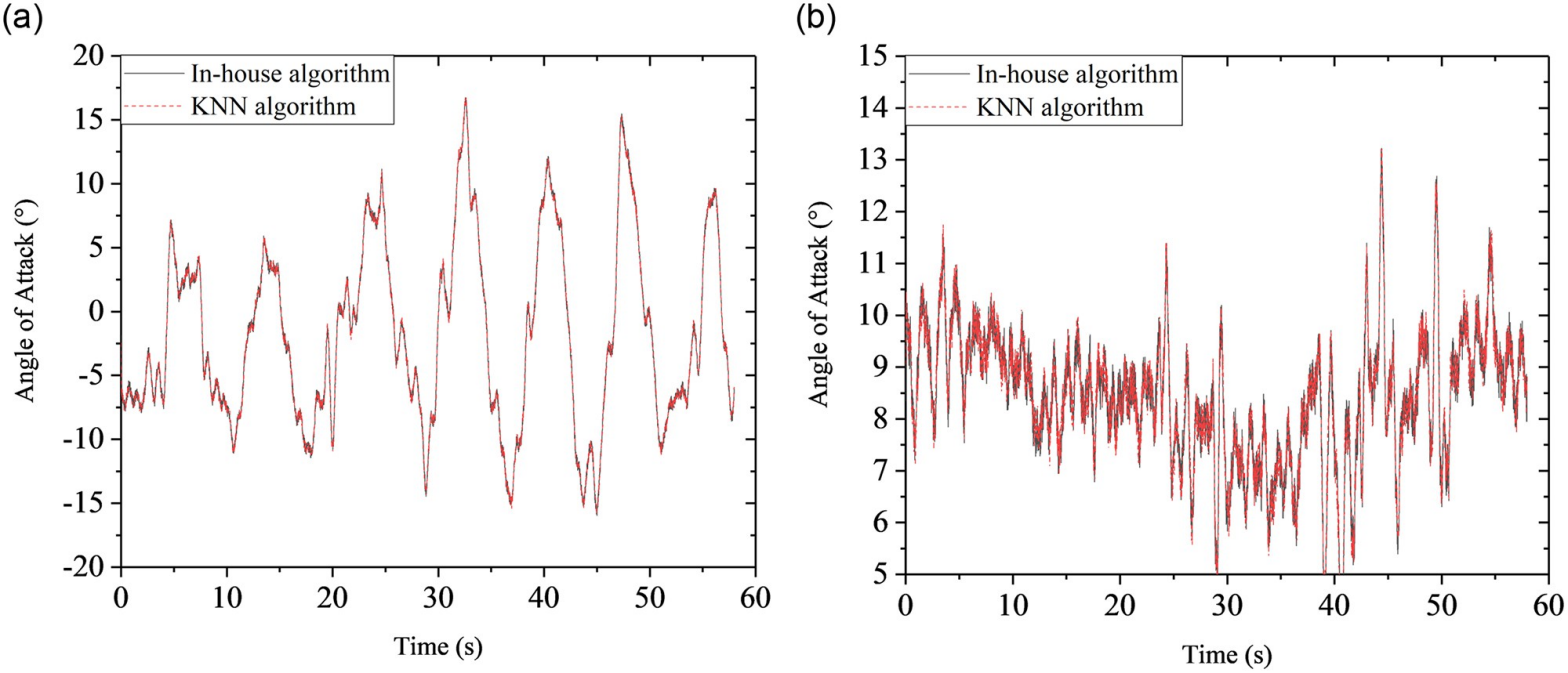
Comparison of angle of attack calculated by in-house algorithm and KNN algorithm. (a) standstill case (b) normal operating case.

Based on a 100kW wind turbine, an aerodynamic measurement system was constructed in the previous research work [6]. In the field test, the flow velocity vector was measured by a self-developed seven-hole probe shown in Fig 31, and the aerodynamic force was obtained with the surface pressure measurement method. It is shown from Fig 30 that the KNN algorithm has the best comprehensive performance. Therefore, in this work, the KNN algorithm is used to calculate the angle of attack of the wind turbine blade in the standstill case and normal operating case. During the data processing, it is found that KNN algorithm is about 100 times faster than the in-house traditional algorithm. Meanwhile, the results from between KNN algorithm and in-house traditional algorithm have good agreement with each other, as shown in Fig 32. In the standstill case, the rotating speed is close to zero, so angle of attack of the blade is dominated by the yawing angle and azimuth angle. Also, the variation range of angle of attack is from about -15° to 15°, which is very large relative to the change of aerodynamic characteristics. In the normal operating case, the rotating speed is higher to make the blade work near the optimal angle of attack. The above example results of field measurements on the angle of attack show that the machine learning method can be more effectively applied to the calibration and measurement of seven-hole probe.

## 7 Conclusions

Machine learning methods have become a popular, convenient and efficient computing tool at present, in which supervised learning mainly solves the data prediction problem. The calibration process of multi-hole probes is exactly the process of using calibration data set to predict the tested results, so machine learning methods are supposed to play a certain advantage role in the calibration of multi-hole probes. In some literatures, specific machine learning method has been developed to apply in the probe calibration, but there is the lack of comprehensive evaluation of machine learning methods. In this work, six typical supervised learning methods in scikit-learn library are selected for parameter adjustment at first. Based on the optimal parameters, a comprehensive evaluation is made from four aspects: prediction accuracy, prediction efficiency, feature sensitivity and robustness on the failure of some hole port. The main conclusions are summarized as follows:

1. Random forests and K-nearest neighbors algorithms have the better comprehensive prediction performance among the six algorithms.
2. Compared with the in-house traditional algorithm, the machine learning algorithms have the great advantages in the computational efficiency and the convenience of writing code.
3. Multi-layer perceptron and support vector machines are the most time-consuming algorithms among the six algorithms.
4. The prediction accuracy of all the algorithms are very sensitive to the features. Using the features based on the physical knowledge can obtain a high accuracy predicted results.
5. The missing of central port data will reduce the prediction accuracy of flow velocity from 1% to about 10% for all the algorithms, and the prediction accuracy of flow angles are abnormal and unacceptable only using polynomial algorithm. The missing of surrounding port data has slight effects on the prediction accuracy for all the algorithms.
6. Due to the ports directly related to sideslip angle are more than the ports related to angle of attack, the prediction accuracy of sideslip angle is always higher than it of angle of attack for all the algorithm.
7. KNN algorithm is applied to field measurements on the angle of attack of a wind turbine blades. It is found that KNN algorithm is about 100 times faster than the in-house traditional algorithm during the data processing, and the results from the two algorithm have good agreements.

The results in this work show that supervised learning methods has great potential in real-time online calibration of multi-hole probes. Nevertheless, some aspects need to be enhanced in further studies. First, the supervised learning methods should be integrated into the online calibration of probes to test real-time. Second, this work is only applied to the 7-hole probe, and can be applied to the n-hole probe in the future. Third, this work mainly discusses the calibration accuracy of pitch angle between -50° and 50°, where port pressure is insensitive to Reynolds number. In the future, the application in larger angle calibration can be analyzed and discussed.

## Supporting information

S1 Appendix (DOCX)

S1 Dataset (XLSX)

## Abbreviations

Cp_i_: pressure coefficient of port pressure
*C*_*q*_, *Cp*_*s*_: pressure coefficient of static pressure
C_α_: pressure coefficient of angle of attack
C_β_: pressure coefficient of sideslip angle
C_ϕ_: pressure coefficient of roll angle
*C*_*o*_, *Cp*_*t*_: pressure coefficient of total pressure
C_θ_: pressure coefficient of pitch angle
P_i_: port pressure
P_s_: static pressure
P_t_: total pressure
U: freestream velocity
u: *x* components of freestream velocity
v: *y* components of freestream velocity
w: *z* components of freestream velocity
x: *x* coordinate
y: *y* coordinate
z: *z* coordinate
α: angle of attack for seven-hole probe
β: sideslip angle for seven-hole probe
ϕ: roll angle for seven-hole probe
θ: pitch angle for seven-hole probe
σ: standard error
