# Supplementary material for: Evaluations on supervised learning methods in the calibration of seven-hole pressure probes
Source: PLoS One. 2023 Jan 23;18(1):e0277672. doi: 10.1371/journal.pone.0277672 (PMC9870118; doi:10.1371/journal.pone.0277672)
Supplement: S1 Appendix — (DOCX) [file pone.0277672.s001.docx]

Appendix: Quantile statistical data table on the prediction errors in Fig.12

Table 1 Quantile statistics on the prediction errors of angle of attack α(°)

| Method | min | quartile 1 | median | quartile 3 | max | RMSE |
| --- | --- | --- | --- | --- | --- | --- |
| KNN | 0 | 0.12067 | 0.27159 | 0.62513 | 5.03571 | 0.66197 |
| Poly | 7.57144E-4 | 0.1436 | 0.38834 | 0.77717 | 5.25038 | 0.78711 |
| MLP | 0.00142 | 0.23856 | 0.44828 | 0.839 | 4.87457 | 0.8211 |
| SVM | 0.00119 | 0.11424 | 0.2992 | 0.67385 | 6.36869 | 0.76916 |
| DTs | 0 | 0 | 0 | 0 | 38.59207 | 1.40997 |
| RFs | 0 | 0.06113 | 0.15345 | 0.3861 | 4.5605 | 0.57095 |
| Traditional | 8.4E-5 | 0.11528 | 0.29799 | 0.71508 | 5.73611 | 0.86 |

Table 2 Quantile statistics on the prediction errors of sideslip angle β(°)

| Method | min | quartile 1 | median | quartile 3 | max | RMSE |
| --- | --- | --- | --- | --- | --- | --- |
| KNN | 0 | 0.08066 | 0.20105 | 0.41401 | 2.76393 | 0.43769 |
| Poly | 5.00352E-4 | 0.10588 | 0.23121 | 0.44242 | 1.93296 | 0.44776 |
| MLP | 0.00202 | 0.13524 | 0.31315 | 0.61262 | 2.54303 | 0.61665 |
| SVM | 1.26315E-5 | 0.09048 | 0.18427 | 0.3919 | 2.1362 | 0.43335 |
| DTs | 0 | 0 | 0 | 0 | 24.30966 | 0.97757 |
| RFs | 0 | 0.06107 | 0.15053 | 0.32494 | 2.61084 | 0.39803 |
| Traditional | 2.41E-4 | 0.09503 | 0.24289 | 0.56185 | 5.66879 | 0.72 |

Table 3 Quantile statistics on the prediction errors of wind speed *v*(%)

| Method | min | quartile 1 | median | quartile 3 | max | RMSE |
| --- | --- | --- | --- | --- | --- | --- |
| KNN | 0.000239485 | 0.106655 | 0.26608 | 0.856755 | 5.30435 | 1.26584 |
| Poly | 0.00054 | 0.11858 | 0.278575 | 1.09572 | 5.43892 | 1.18131 |
| MLP | 0.00064 | 0.12806 | 0.287325 | 1.335435 | 7.71722 | 1.564445 |
| SVM | 0.000905 | 0.118615 | 0.285695 | 1.189945 | 4.813185 | 1.21353 |
| DTs | 0.000745 | 0.14163 | 0.349125 | 0.754485 | 11.50394 | 1.139265 |
| RFs | 0.000248146 | 0.112055 | 0.28003 | 0.825755 | 4.82862 | 1.019615 |
| Traditional | 0.00005 | 0.21575 | 0.4603 | 0.91395 | 6.46545 | 0.97 |
